# Photothermal Heating and Real‐Time In Situ Luminescent Thermometry with Iron Oxide Core‐Silica Shell Nano‐Objects

**DOI:** 10.1002/smll.202508497

**Published:** 2025-10-22

**Authors:** Farah Abdel Sater, Gautier Félix, Saad Sene, Udara Bimendra Gunatilake, Basile Bouvet, Tristan Pelluau, Erwan Oliviero, Albano Neto Carneiro Neto, Luís Dias Carlos, Belén Albela, Laurent Bonneviot, Yannick Guari, Joulia Larionova

**Affiliations:** ^1^ ICGM Univ. Montpellier, CNRS, ENSCM route de Mende CNRS Occitanie Est, 1919 Montpellier 34293 France; ^2^ Department of Physics and CICECO – Aveiro Institute of Materials University of Aveiro Aveiro 3810‐193 Portugal; ^3^ Max‐Planck‐Institut für Kohlenforschung Kaiser‐Wilhelm‐Platz 1 D‐45470 Mülheim an der Ruhr Germany; ^4^ Laboratoire de Chimie ENS de Lyon Université de Lyon Lyon 69364 France

**Keywords:** iron oxide nanoparticles, multifunctional nanoparticles, photothermia, real‐time temperature detection, thermometry

## Abstract

In a world increasingly focused on precision and efficiency, achieving reliable nanoscale thermal feedback in nanoparticle‐assisted heating presents significant challenges. It requires meticulous control over the morphology of nano‐objects and the precise spatial arrangement of the heater and the local temperature probe, both of which are critical for accurate surface temperature readouts. In this work, real‐time nanoscale temperature measurements are presented during the nanoparticle‐assisted photothermal heating by using new nano‐objects consisting of single iron oxide nanoparticles encased in stellate‐like silica shells, loaded with a luminescent coordination compound, namely [(Tb/Eu)_9_(acac)_16_(μ_3_‐OH)_8_(μ_4_‐O)(μ_4_‐OH)]. These multifunctional nano‐objects act as efficient light‐triggered nano‐heaters and as ratiometric luminescent thermometers operating between 20–65 °C in water with excellent cyclability and good maximum relative thermal sensitivity of 0.75 ± 0.02% °C^−1^ at 65 °C and thermal uncertainty of 1 °C. Real‐time in situ temperature monitoring *via* the Tb^3+^/Eu^3+^ luminescence intensity ratio during photothermal heating under 808 nm irradiation demonstrates reproducibility and reliable thermal feedback, highlighting its potential for advanced temperature‐responsive applications.

## Graphical abstract

Real‐time in‐situ operando thermal feedback during photothermal heating in water is achieved using multifunctional nano‐objects composed of iron oxide cores enwrapped in stellate‐like silica shells and loaded with luminescent Tb^3^⁺/Eu^3^⁺ thermometers, offering high sensitivity, reproducibility, and precise nanoscale temperature control.

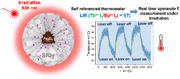

## 1 Introduction

Inorganic nano‐objects capable of generating significant heat when remotely activated by external stimuli have garnered considerable attention over the past several decades. This increasing interest is attributed to their promising applications across various fields, including hyperthermia treatment of cancer^[^ ^1^, ^2^, ^3^, ^4^ ^]^ and bacteria‐related diseases,^[^ ^5^, ^6^, ^7^, ^8^, ^9^, ^10^, ^11^ ^]^ drug and gene delivery,^[^ ^12^, ^13^, ^14^, ^15^, ^16^, ^17^ ^]^ low‐temperature catalysis,^[^ ^18^ ^]^ regulation of enzymatic reactions,^[^ ^19^ ^]^ and others. For this reason, numerous nano‐heaters activated either by light irradiation (photothermal agents) or by the application of an alternating current magnetic field (magnetothermal agents), have been extensively reported.

A major challenge in nanoparticle‐assisted heat generation is achieving precise temperature control, particularly at the nanoscale surface/interface. This is crucial not only for understanding nanoscale heat transfer but also for improving current applications and enabling new ones. However, most reported studies rely on macroscopic temperature measurements using conventional probes, which fail to capture nanoscale variations. Recent findings suggest that heat transfer at the nanoscale deviates from macroscopic laws, yet experimental limitations make it difficult to verify.^[^ ^20^ ^]^ In this context, the concept of “hot nano‐spots” or “thermal inhomogeneity” in nanoparticle‐assisted heating has emerged.^[^ ^14^ ^]^ This idea proposes that a significant temperature gradient may exist between nanoparticles activated by external stimuli and their surrounding macroscopic environment.^[^ ^18^, ^21^, ^22^, ^23^, ^24^ ^]^ While still debated,^[^ ^25^, ^26^, ^27^ ^]^ this concept challenges conventional heat diffusion theories and enables breakthroughs, especially when bulk heating is ineffective.

This promise has boosted the development of various nanoscale temperature sensing methods able to work in combination with magnetothermal or photothermal heating.^[^ ^28^, ^29^, ^30^, ^31^, ^32^, ^33^ ^]^ Among them, the most embraced approach consists of employing photoluminescent thermometry based on the temperature‐dependent emission of different luminescent species.^[^ ^34^, ^35^, ^36^, ^37^, ^38^, ^39^, ^40^, ^41^, ^42^, ^43^ ^]^ Early luminescent thermometry during photothermal heating mainly used plasmonic gold heaters with electrostatically attached sensors,^[^ ^44^, ^45^, ^46^, ^47^, ^48^, ^49^, ^50^, ^51^ ^]^ an approach that suffered from components leaching and limited control over sensor–heater positioning. The drawbacks have been partially addressed using lanthanide fluorides,^[^ ^52^, ^53^, ^54^, ^55^ ^]^ oxifluoride,^[^ ^56^ ^]^ or Ag_2_S^[^ ^41^, ^57^ ^]^ nanoparticles, which enable temperature elevation under near‐infrared (NIR) irradiation, while exhibiting self‐monitored luminescence for temperature sensing. Despite good size and shape control, these nanostructures still suffer from low photothermal or photoluminescence efficiency, stability issues, broad emission, and potential photodegradation.

Alternatively, iron oxide nanoparticles (IONPs) have recently been reported as efficient photothermal agents under NIR irradiation, leveraging their absorption and significant light‐to‐heat conversion to generate macroscopic heat.^[^ ^58^, ^59^, ^60^, ^61^, ^62^, ^63^, ^64^, ^65^ ^]^ Moreover, IONPs offer size and shape tunability, easy surface functionalization, and biocompatibility.^[^ ^2^, ^66^, ^67^, ^68^ ^]^ Surprisingly, nanoscale heating around IONPs during photothermia remains largely unexplored, though one study reported IONP core temperature elevation relative to the macroscopic temperature, inferred from enhanced IONP degradation under light irradiation.^[^ ^33^ ^]^ Note that IONP nano‐systems developed for temperature monitoring during magnetothermia are not well suited for simultaneous photothermal heating and temperature sensing due to the inherent limitations of their components. First, some systems incorporate organic fluorophores,^[^ ^22^, ^25^ ^]^ or proteins,^[^ ^23^ ^]^ which suffer from low photothermal stability and broad overlapping emission bands, limiting the reliability of luminescence intensity ratio (LIR)‐based thermometry. Moreover, polymer shells enwrapping luminescent temperature probes, such as organic dyes^[^ ^24^, ^38^, ^69^ ^]^ or Ln^3+^ complexes,^[^ ^34^, ^70^ ^]^ exhibit temperature‐dependent softening, viscosity changes, and low thermal conductivity, which reduce the accuracy of temperature readouts. Beyond material limitations, achieving reliable temperature measurement demands precise morphological control of the nanostructure. This entails a well‐defined, single heater core in close proximity to a uniformly distributed luminescent thermometer, while minimizing the risk of component leaching.

In this article, we report real‐time in situ temperature detection during a photothermal process using new well‐defined nano‐objects composed of a single IONP heater core enwrapped by a controlled stellate‐like silica shell containing a lanthanide‐based luminescent thermometer. In contrast to polymers, the silica shell provides a rigid, thermally stable, and optically transparent matrix, ensuring precise spatial arrangement of the luminescent thermometer and uniform heat distribution suitable for emissive temperature measurements during photothermal heating. Despite an effort in the synthesis of IONPs enwrapped by silica shells,^[^ ^71^, ^72^, ^73^, ^74^, ^75^, ^76^ ^]^ they have scarcely been employed for the design of heater/thermometer nanoparticles.^[^ ^42^, ^77^ ^]^ We selected in this work a stellate‐like silica shell for its tunable large‐pore structure enabling loading of bulky luminescent complexes, open center‐radial pore morphology allowing optimized diffusion/mass transport, good thermal shock resistance with minimal expansion, and optical transparency for light activation and thermometry.^[^ ^75^, ^78^, ^79^, ^80^, ^81^ ^]^ We selected the [(Tb/Eu)_9_(acac)_16_(μ_3_‐OH)_8_(μ_4_‐O)(μ_4_‐OH)] (acac = acetylacetonate) complex as a ratiometric luminescent thermometer based on its excellent temperature‐sensing performance after encapsulation.^[^ ^82^, ^83^ ^]^ Therefore, we coated the IONP core with a functionalized stellate‐like silica shell and loaded it with the luminescent [(Tb/Eu)_9_(acac)_16_(μ_3_‐OH)_8_(μ_4_‐O)(μ_4_‐OH)]·H_2_O complex. The resulting nano‐objects present a well‐defined morphology with a single IONP core surrounded by a silica shell with a uniform complex distribution, functioning as efficient nano‐heaters triggered by 808 nm irradiation. They exhibit temperature‐dependent Tb^3+^ and Eu^3+^ luminescence, with an exceptionally high Tb^3+^→Eu^3+^ energy transfer rate in the order of 10^11^ s^−1^.^[^ ^84^, ^85^, ^86^ ^]^ The best energy transfer‐driven nanothermometer operates in the 20–65 °C range in water with good cyclability and maximum relative thermal sensitivity of 0.75 ± 0.02% °C^−1^ at 65 °C and thermal uncertainty of 1 °C. The thermal feedback reliability from these nano‐objects was confirmed by real‐time temperature in situ monitoring during the photothermal heating *via* the Tb^3+^/Eu^3+^ LIR.

## 2 Results and Discussion

### 2.1 Design of IONP Core@SiO_2_ Shell Nano‐Objects Incorporated Luminescent Tb^3+^/Eu^3+^ Complex

**Scheme 1:**
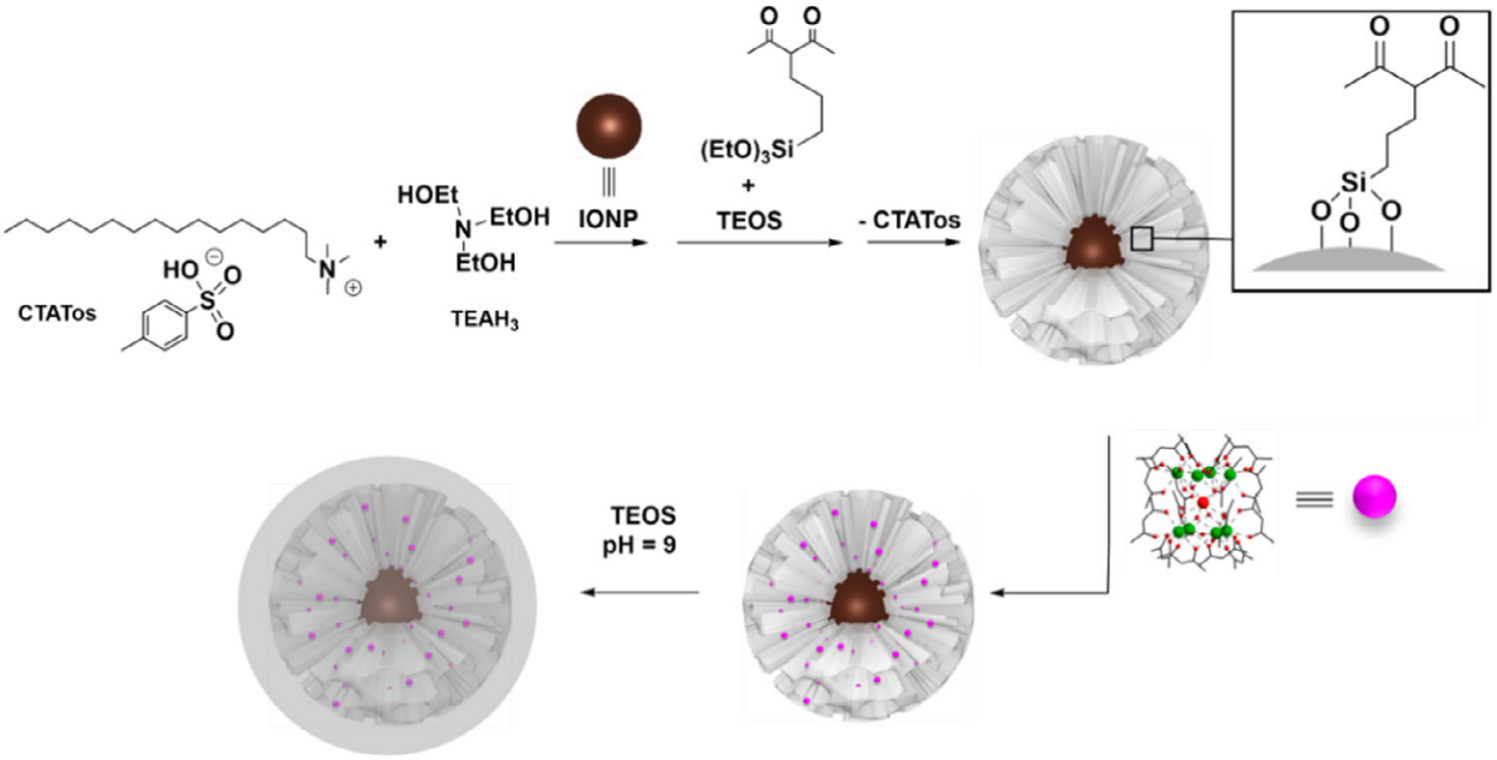
Representation of the synthesis of multifunctional core@shell IONP@SiO_2_‐acac nanoparticles loaded with the luminescent [(Tb/Eu)_9_(acac)_16_(μ_3_‐OH)_8_(μ_4_‐O)(μ_4_‐OH)] complex.

The synthesis of multifunctional core@shell IONP@SiO_2_ nanoparticles, loaded with a luminescent [(Tb/Eu)_9_(acac)_16_(μ_3_‐OH)_8_(μ_4_‐O)(μ_4_‐OH)] compound within the silica shell, was performed using a four‐step approach (**Scheme** **1**). It involves: i) synthesizing the pristine IONP nanoparticles stabilized by oleate and oleylamine, ii) growing a mesoporous silica shell functionalized with acetylacetonate on the surface of IONP, iii) incorporating the luminescent compound into the shell's porosity, and iv) sealing the pores.

The synthesis of pristine IONPs was performed through a thermal decomposition method, followed by an oxidation step to convert the majority of FeO into Fe_3_O_4_, as previously reported.^[^ ^87^ ^]^ The functionalized mesoporous silica shell was grown on the surface of the IONP using an adapted sol–gel procedure.^[^ ^39^ ^]^ The silica pores functionalization with acac was carried out in situ through a co‐condensation reaction during the sol–gel reaction by adding an acac‐siloxane derivative and TEOS. The acac functionalization of the silica pores is essential, as complex encapsulation is inefficient with a non‐functionalized silica.

The pH variation during the silica shell formation allowed two stellate‐like morphologies of IONP@SiO_2_‐acac nanoparticles with different pores opening, (**1A**) at pH = 7 and (**1B**) at pH = 5.6 (see Supporting Information). The morphological change can be attributed to the competitive adsorption of the tosylate anion from the surfactant and silicate oligomers on micelles.^[^ ^80^, ^88^ ^]^ After nanoparticles’ formation, the surfactant was removed through washings, which opened up the silica porosity and allowed access to the acetylacetonate groups (see **Table** **1** for main characteristics of **1A** and **1B**). Next, the [(Tb/Eu)_9_(acac)_16_(μ_3_‐OH)_8_(μ_4_‐O)(μ_4_‐OH)]·H_2_O compound with a Tb/Eu ratio of 9/1 was inserted into the pores of the silica shells resulting in samples **2A** and **2B**. Finally, the pores were sealed using TEOS to trap the inserted complex inside them, preventing its leaching. The obtained samples were labeled as **3A** and **3B** for respectively less and more open stellate‐like porosity of the silica shell. EDS (Fe, Si, Tb, and Eu) and ICP (Fe, Tb) analyses were performed to ascertain the chemical formula of samples **1A**, **B** – **3A**, **B** (see Experimental Part, Supporting Information, Table 1). Samples **3A** and **3B** presented a loading capacity of 0.0023 and 0.0083 units of complex per SiO_2_ unit, equivalent to 0.021 and 0.075 Tb/Eu units per unit of SiO_2_, respectively. Note that the more open morphology of the silica shell in **3B** enhances complex insertion, increasing the loading capacity by more than 3.6 times. Note that a comparable loading was observed in our previous work.^[^ ^82^ ^]^ Infrared (IR) spectroscopy confirms the successful grafting of the acac functions into the silica pores for samples **1A** and **1B**, as well as the [(Tb/Eu)_9_(acac)_16_(μ_3_‐OH)_8_(μ_4_‐O)(μ_4_‐OH)] complex loading for **3A** and **3B** (Figure S1, Supporting Information).

**Table 1:** Some characteristics of samples **1A, B** – **3A, B**.

	Composition^a)^	*d*_core_ [nm]	*d*_whole_ [nm]	*d*_shell_ [nm]
**1A**	IONP_0.109_@(SiO_2_)_1_(CTA)_0.0123_(acac)_0.0296_	25.6 ± 0.8	103.7 ± 4.7	38.9 ± 2.4
**1B**	IONP_0.123_@(SiO_2_)_1_(CTA)_0.0127_(acac)_0.0302_	25.8 ± 0.9	93.1 ± 4.6	33.6 ± 2.4
**2A** **2B** **3A** **3B**	IONP_0.109_@(SiO_2_)_1_(CTA)_0.0123_(acac)_0.0296_/(Tb/Eu)_0.0041_ IONP_0.123_@(SiO_2_)_1_(CTA)_0.0127_(acac)_0.0302_/(Tb/Eu)_0.0227_ IONP_0.109_@(SiO_2_)_0.963_(CTA)_0.0123_(acac)_0.0296_/(Tb/Eu)_0.0023_@SiO_0.037_ IONP_0.123_@(SiO_2_)_0.965_(CTA)_0.0127_(acac‐Si)_0.0302_/(Tb/Eu)_0.0083_@SiO_0.035_	25.7 ± 0.8 25.5 ± 0.9 25.6 ± 0.8 25.6 ± 0.8	103.1 ± 1.8 93.8 ± 3.7 109.8 ± 4.3 107.3 ± 5.2	38.6 ± 1.1 33.9 ± 1.9 41.9 ± 2.2 40.7 ± 2.1

^a)^ Calculated from ICP and EDS.

**Figure 1:**
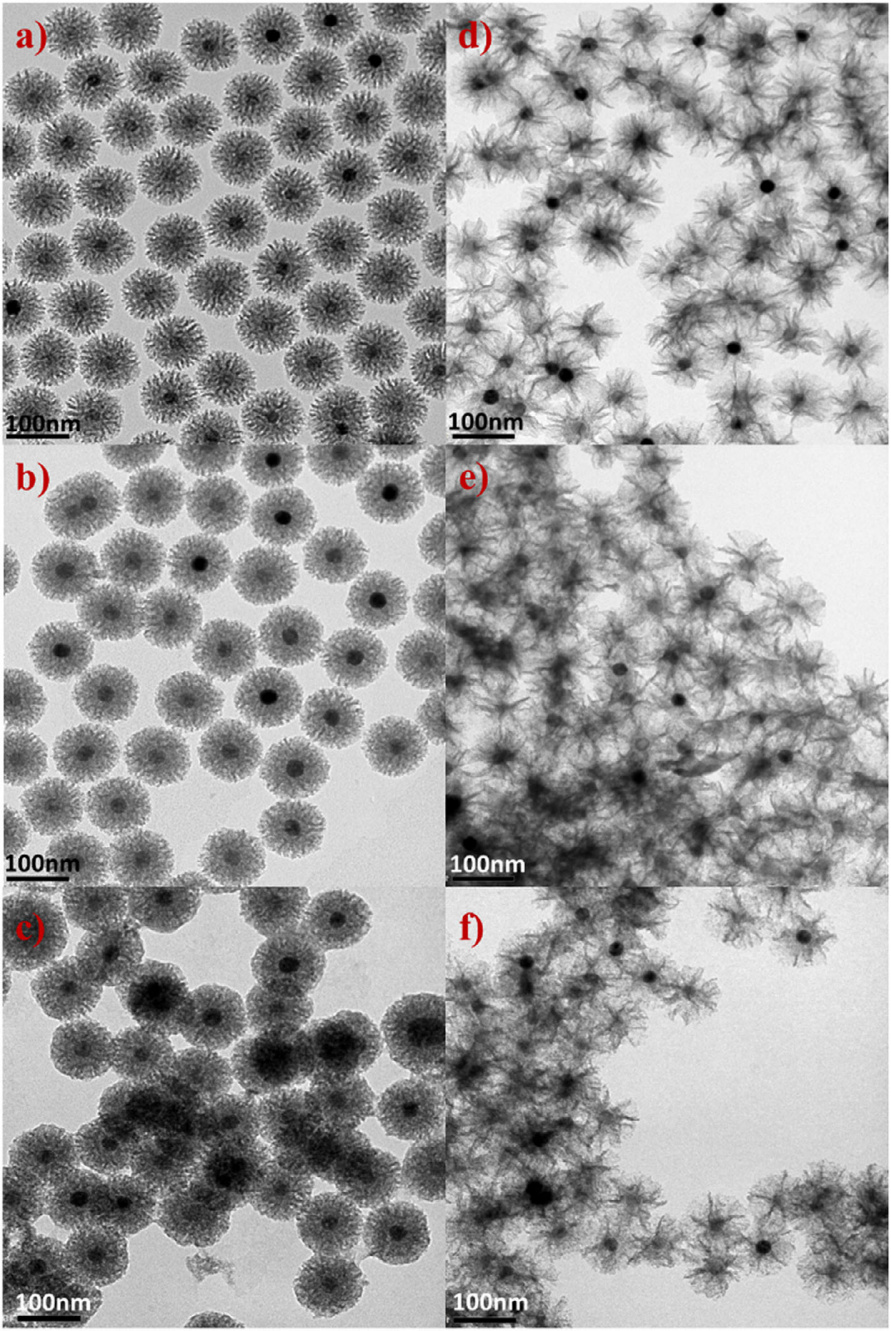
TEM images of: core@shell IONP@SiO_2_‐*acac* nanoparticles **1A** (a) and **1B** (b); IONP@SiO_2_‐*acac*/(Tb/Eu)_9_ nanoparticles **2A** (c) and **2B** (d); IONP@SiO_2_‐*acac*/(Tb/Eu)_9_@SiO_2_ nanoparticles **3A** (e) and **3B** (f). Scale bar = 100 nm.

Transmission electron microscopy (TEM) images reveal that IONP@SiO_2_‐*acac* nanoparticles **1A** and **1B** exhibit a core–shell morphology with a single spherical IONP core of 25.6 ± 0.8 nm coated with a stellate‐like silica shell (**Figure** **1**). The spherical morphology and size of the IONP core are preserved following shell growth, as pristine IONP have the size of 25.9 ± 0.9 nm (Figures S2 and S3, Supporting Information). Both silica shells possess open radial porosity with less or more pronounced openings observed for **1A** and **1B**, respectively. The overall nanoparticle sizes are 103.7 ± 4.7 nm for **1A** and 93.1 ± 4.6 nm for **1B** (Figure 1; Figure S3, Supporting Information, Table 1). Therefore, the silica shell thicknesses, calculated as the difference between the overall size and the IONP core size, are equal to 41.9 ± 2.2 and 40.7 ± 2.1 nm for **1A** and **1B**, respectively (Table 1). Nitrogen sorption isotherms confirm the open porosity of the silica shell in **1A** and the successful pore clogging for **3B** (Figure S4, Supporting Information). The obtained specific surfaces decrease from 418.22 to 71 m^2^ g^−1^ for **1A** and **3B,** respectively.

Incorporation of the [(Tb/Eu)_9_(acac)_16_(μ_3_‐OH)_8_(μ_4_‐O)(μ_4_‐OH)] complex into the silica shell porosity did not significantly alter the nanoparticle size (Figures S1 and S3, Supporting Information, Table 1). On the other hand, silica pore clogging increased the overall nanoparticle size (109.8 ± 4.3 and 107.3 ± 5.2 nm for **3A** and **3B**, respectively), while IONP core size remained unchanged (Figures S1 and S3, Supporting Information, Table 1). This fact, along with a slight modification of porosity, particularly in the more obstructed stellate morphology (**3A**), suggests successful pore clogging. Dynamic light scattering measurements in water indicate that the nanoparticles are well dispersed and non‐aggregated (Figure S5, Supporting Information).

**Figure 2:**
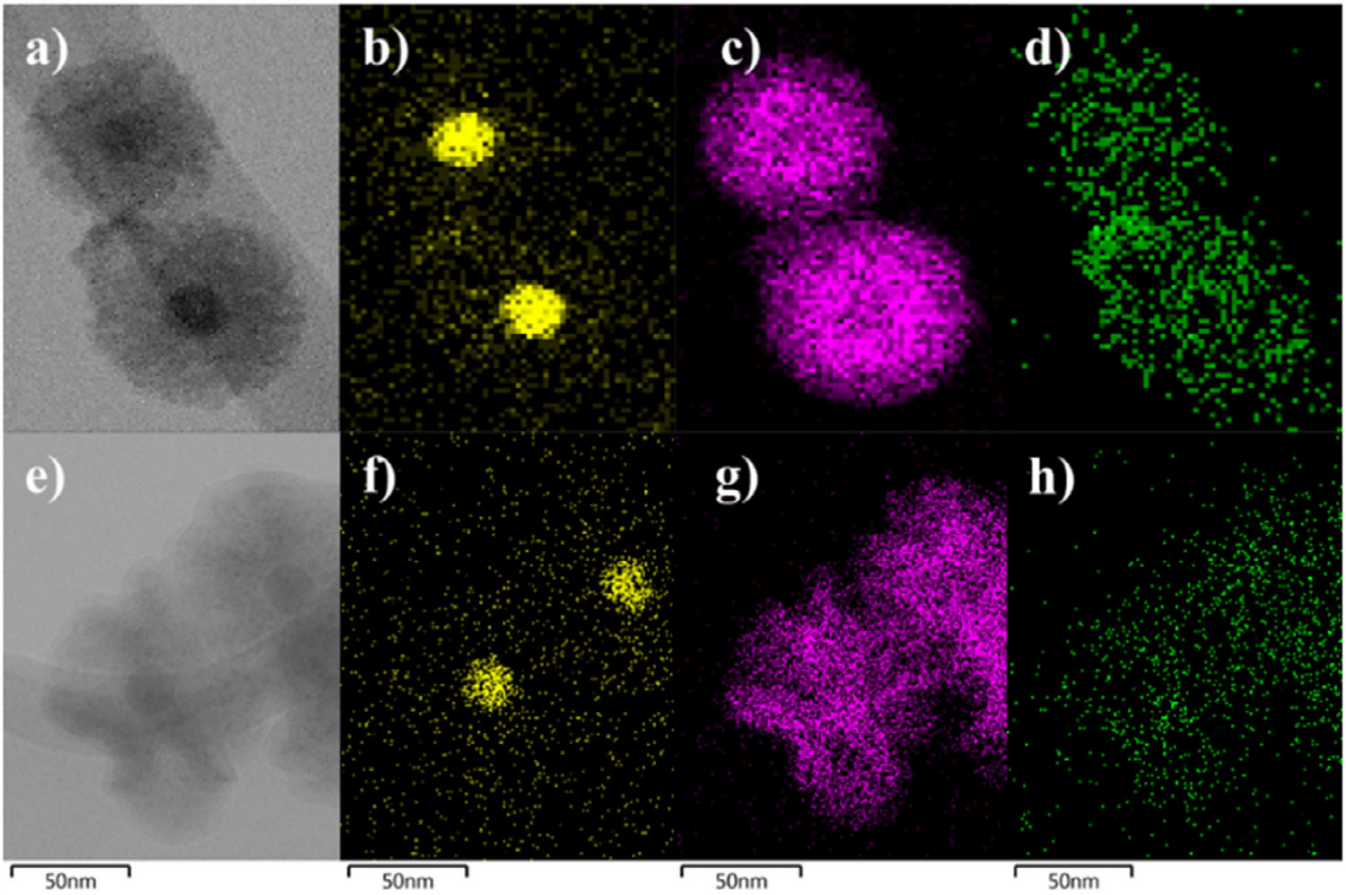
STEM‐bright field images (a,e) and STEM‐EDS elemental mapping of IONP@SiO_2_‐*acac*/(Tb/Eu)_9_@SiO_2_ nanoparticles **3A** (top) and **3B** (bottom) with topochemical distribution of Fe (yellow) (b,f), Si (pink) (c).

The IONP@SiO_2_‐acac/(Tb/Eu)_9_@SiO_2_ nanoparticles were characterized using HAADF‐STEM with EDS mapping, which confirmed their core@shell morphology (**Figure** **2**). The iron atoms from IONP are depicted in yellow, surrounded by a silicon shell from SiO_2_ shown in pink. The terbium atoms (in green) are homogeneously distributed within the silica shell, confirming the successful encapsulation of the complex. Note that the europium atoms could not be clearly visualized due to their very low amount, a limitation previously reported by another group^[^ ^70^ ^]^ and in our previous work.^[^ ^82^ ^]^

### 2.2 Photothermal Properties

**Figure 3:**
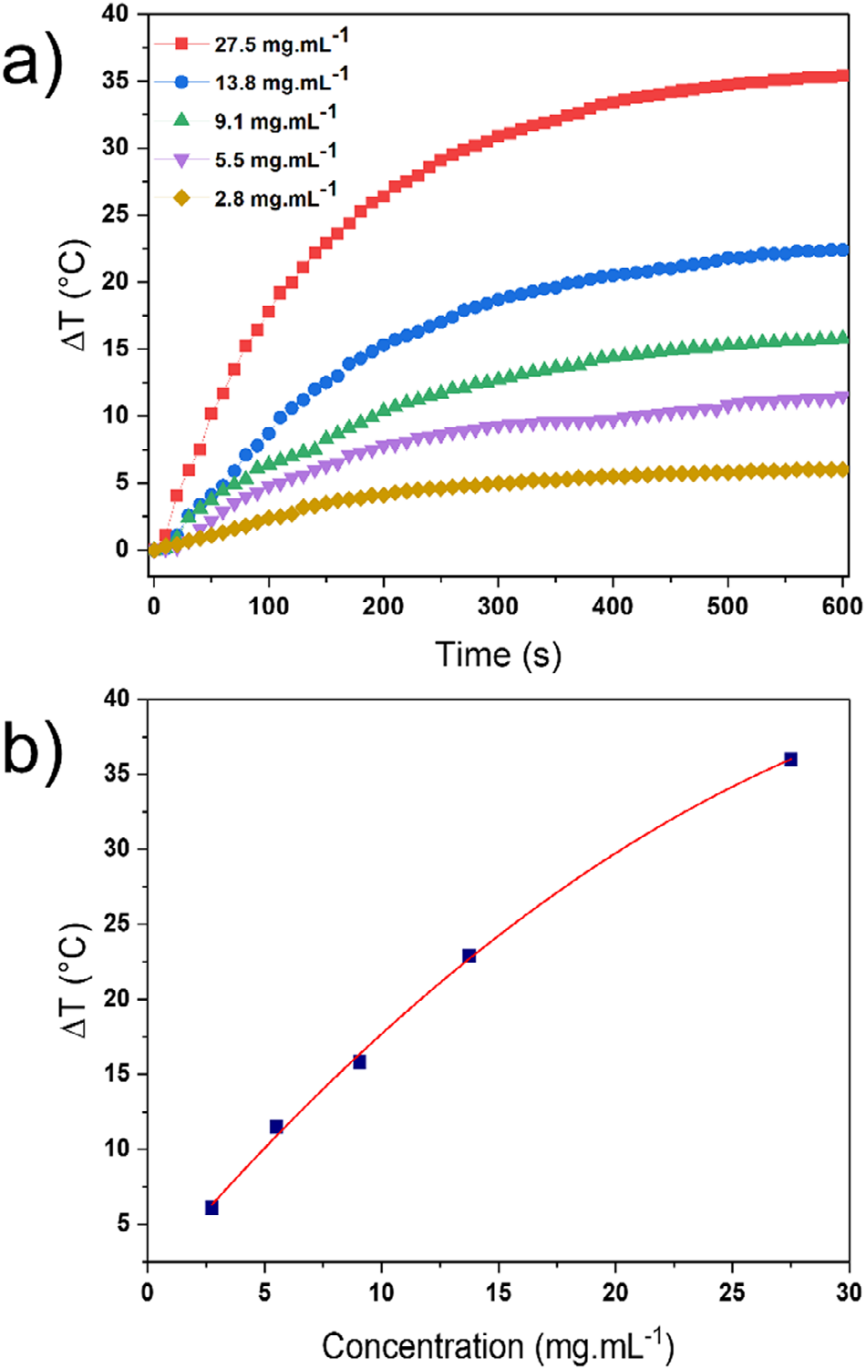
a) Δ*T* versus time curves performed for aqueous colloidal solutions of **3A** with different concentrations of IONP (taken as Fe_3_O_4_) performed under irradiation at 808 nm (2.58 W cm^−2^) with *ΔT* representing the difference between the temperatures of colloidal solutions of **3A** and water; b) Maximal temperature achieved at 10 min as a function of IONP concentration for **3A**.

While IONPs have long been recognized as effective magnetothermal heaters, their photothermal properties have only attracted significant attention over the past decade.^[^ ^58^, ^59^, ^60^, ^61^, ^62^, ^63^, ^90^, ^91^, ^92^ ^]^ However, the mechanisms underlying their radiation‐to‐heat conversion are not yet fully understood.^[^ ^62^, ^93^, ^94^ ^]^ It has been demonstrated that, unlike magnetothermia, photothermal heating primarily depends on laser power and solvent properties and is only minimally influenced by the size and shape of IONP.^[^ ^95^ ^]^ While several studies have explored the photothermal properties of IONP core‐silica shell nanoparticles with either dense^[^ ^72^, ^96^ ^]^ or dendritic silica shells,^[^ ^39^, ^75^ ^]^ comparison between them and uncoated IONPs with the same core remains rather controversial. The photothermal effect of nanoparticles **3A** and **3B** in water was investigated under 808 nm laser irradiation at a power of 2.58 W cm^−2^. A significant temperature increase dependent on nanoparticle concentration was observed for both samples after 10 min irradiation, while no noticeable heating was detected in water (Δ*T* = 0.1 °C) (**Figure** **3**a; Figure S6, Supporting Information). Figure 3a shows Δ*T* versus time measurements for colloidal solutions of sample 3A at various concentrations from 2.8 to 27.5 mg mL^−1^ of IONP (taken as Fe_3_O_4_) (or from 0.03 to 0.35 mol L^−1^ of [Fe]), where Δ*T* represents the temperature difference between the nanoparticles’ solution and water. The Δ*T* value increases linearly with time during the first two minutes and then tends to saturation. The curve Δ*T*_max_ (reached after ten minutes) versus IONP concentration plotted in Figure 3b indicates that macroscopic heating varies linearly at low concentrations and then deviates from linearity for the higher ones due to the limited penetration depth of NIR light, affected by absorption and diffusion phenomena. The macroscopic photothermal properties of these nanoparticles are comparable to those of IONPs coated with gallic acid, which share the same IONP core but lack a silica shell, obtained under similar conditions (Figure S7, Supporting Information). This result suggests that the stellate‐like silica shell with open porosity does not significantly impede heat transfer from the IONP core to the surrounding water. Moreover, the preservation of photothermal efficiency indicates that the shell is thermally permeable at the nanoscale, acting as a protective and functional scaffold without compromising the core's heating capability. Note also that the obtained heating for our **3A** and **3B** nanoparticles falls in the range of results previously obtained for IONP core stellate‐like shell nanoparticles, although those studies involved different IONP composition and were conducted under different conditions (irradiation at 1064 with the laser power of 1–2 W cm^−1^).^[^ ^39^ ^]^

The photothermal conversion efficiency, *η*, was calculated by fitting the *T* versus time curves with the COMSOL software^[^ ^97^ ^]^ (see Figure S6, Supporting Information for the fits and Section 2.4, Supporting Information for details). The obtained *η*‐value stands at 89 ± 1 and 84 ± 1% for **3A** and **3B**, respectively. Although these values should be interpreted with caution since different estimation methods and experimental conditions can provide varying results,^[^ ^98^ ^]^ they are nonetheless among the highest reported for IONP‐based systems.^[^ ^65^ ^]^

### 2.3 Photoluminescence and Luminescence Thermometry

**Figure 4:**
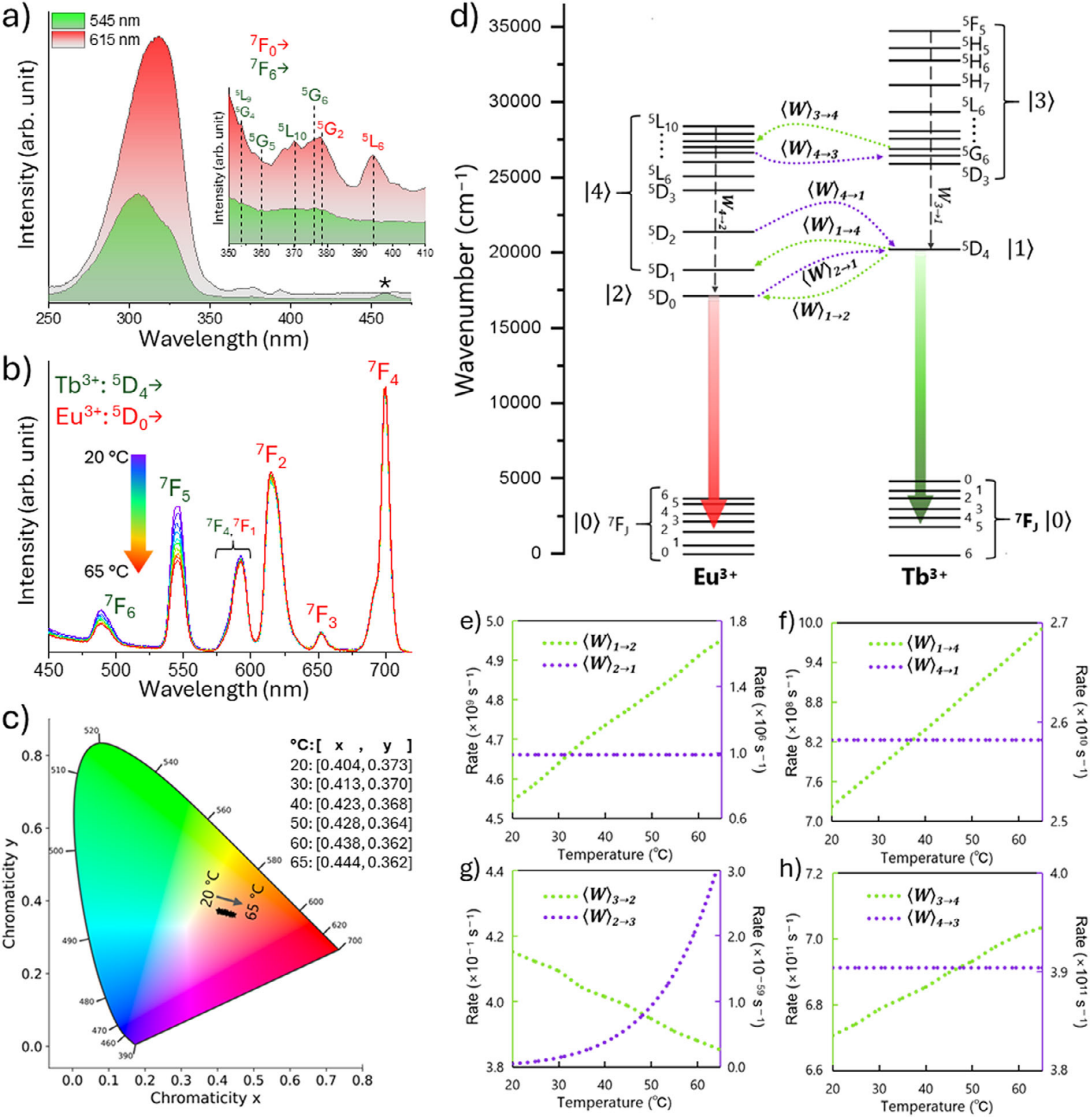
a) Room temperature excitation spectra of **3A** in water, monitored at *λ*_em_ = 545 nm (green) and 615 nm (red); the asterisk denotes the Raman scattering peak of water). b) Temperature‐dependent emission spectra (20–65 °C) of **3A** in water under 315 nm excitation, showing quenching of the Tb^3+ 5^D_4_→^7^F_J_ transitions. c) CIE chromaticity coordinates illustrating the red shift of emission color with increasing temperature; d) Energy level diagram indicating forward and backward energy transfer; e–h) Temperature dependence of the calculated energy transfer rates ${\langle W\rangle }_{i\to j}$: forward rates (i odd → j even) and backward rates (i even → j odd).

While the [(Tb/Eu)_9_(acac)_16_(μ_3_‐OH)_8_(μ_4_‐O)(μ_4_‐OH)] complex is not water‐soluble, its encapsulation within the silica shell of IONP@SiO_2_‐acac/(Tb/Eu)_9_@SiO_2_ nanoparticles enables the observation of luminescence in aqueous colloidal solutions. First, the luminescent properties of samples **3A** and **3B** were investigated in water at pH = 7.4 to confirm the successful incorporation of the complex within the silica shell's porosity. The photophysical behavior and energy transfer dynamics of **3A** and **3B** in aqueous suspension in the 20–65 °C temperature range are demonstrated in **Figure** **4** and Figure S8 (Supporting Information). The excitation spectra monitoring the Tb^3+ 5^D_4_→^7^F_5_ (green curve) and Eu^3+ 5^D_0_→^7^F_2_ (red curve) show broad bands between 250 and 350 nm, attributed to the sensitization of the Ln^3+^ from the acac ligand (Figure 4a; Figure S8a, Supporting Information). The inset of Figure 4a magnifies the region from 350 to 410 nm, revealing characteristic Tb^3+^ transitions (e.g., ^7^F_6_→^5^G_5_, ^7^F_6_→^5^L_10_, and ^7^F_6_→^5^G_6_) when the emission of the Eu^3+^ is monitored (red curve), thereby confirming efficient Tb^3+^ → Eu^3+^ energy transfer. The energy transfer is further evidenced in the excitation spectrum of **3B**, monitored at 700 nm (Eu^3+ 5^D_0_ → ^7^F_4_ transition), where a band at 490 nm, assigned to the Tb^3+ 7^F_6_→^5^D_4_ transition, and another at 380 nm, corresponding to the Tb^3+ 7^F_6_→^5^G_6_, ^5^D_3_ transitions, are observed (Figure S8a, Supporting Information). The emission spectra of **3A** and **3B** under 315 nm excitation exhibit a series of characteristic transitions for Tb^3+ 5^D_4_→^7^F_6‐0_ and Eu^3+ 5^D_0_→^7^F_0‐4_ (Figure 4b; Figure S8b, Supporting Information, in green and red labels, respectively). Similar transitions were observed in the spectra of [(Tb/Eu)_9_(acac)_16_(μ_3_‐OH)_8_(μ_4_‐O)(μ_4_‐OH)]·H_2_O (Figure S9, Supporting Information) proving its successful encapsulation. However, several modifications can be noted in the relative intensities of the ^5^D_4_→^7^F_5_/^5^D_0_→^7^F_2_ and the ^5^D_4_→^7^F_5_/^5^D_0_→^7^F_4_ emissions when the spectra of **3A** and **3B** are compared with the free complex. In fact, for **3A**, the Tb^3+^/Eu^3+^ intensity ratio of the ^5^D_4_→^7^F_5_/^5^D_0_→^7^F_2_ and the ^5^D_4_→^7^F_5_/^5^D_0_→^7^F_4_ transitions went from 3.8 and 22 to 0.72 and 0.47 after encapsulation. A similar tendency was observed in the case of **3B** (Figure S8f, Supporting Information), for which the intensity ratios ^5^D_4_→^7^F_5_/^5^D_0_→^7^F_2_ and ^5^D_4_→^7^F_5_/^5^D_0_→^7^F_4_ were found to be equal to 0.83 and 0.63 after encapsulation. This dramatic change in the relative intensities of the Ln^3+^ transitions was previously observed in dendritic silica nanoparticles containing the same complex and explained by theoretical modeling.^32^ Encapsulation of the complex within the functionalized silica pores was shown to induce slight symmetry modifications, shorten intermetallic Ln^3+^–Ln^3+^ distances, and thereby enhance Tb^3+^ to Eu^3+^ energy transfer, which is responsible for the thermometric performance, as we will show from simulations. Moreover, the absorption from the IONP core can also impact the emission spectrum in the visible region.

Second, the temperature‐dependent luminescence of nanoparticles **3A** and **3B** was investigated to evaluate their employment as a luminescent thermometer in water. While the peak positions in the emission spectra of both samples remain fixed, indicating that there is no energy shifting, the intensity of the Tb^3+ 5^D_4_→^7^F_J_ (*J* = 6, 5, and 4) transitions steadily reduces as temperature rises, reflecting thermally enhanced Tb^3+^→Eu^3+^ energy transfer (Figure 4b; Figure S10, Supporting Information). The CIE chromaticity diagram for **3A** shown in Figure 4c exhibits a continuous red shift with increasing temperature, highlighting the growing Eu^3+^ participation to the overall emission due to the enhanced energy transfer, despite a Tb^3+^/Eu^3+^ ratio of 9:1 in the compound. Figure 4d presents the adopted energy level diagram for the (Tb/Eu)_9_ cluster, with arrows denoting the forward energy transfer rates ${\langle W\rangle }_{odd\to even}$ and corresponding backward rates, ${\langle W\rangle }_{even\to odd}$. The temperature‐dependent energy transfer rates, computed via state‐of‐the‐art theoretical models (see Supporting Information for further detail),^[^ ^84^, ^85^, ^99^ ^]^ are plotted in Figure 4e–h. In Figure 4e, the forward transfer involving transitions from the Tb^3+ 5^D_4_ and Eu^3+ 5^D_0_ emitting levels ($\left({\langle W\rangle }_{1\to 2}\right)$), besides being more than three orders of magnitude higher than its back transfer (${\langle W\rangle }_{2\to 1}$), it steadily increases with temperature, whereas its reverse remains almost invariant. This is direct evidence of the quenching of the Tb^3+ 5^D_4_→^7^F_J_ emissions observed in Figure 4b. A similar trend appears for the higher‐lying channel ${\langle W\rangle }_{1\to 4}$ and ${\langle W\rangle }_{3\to 4}$ in Figure 4f,h, contributing to the overall enhanced Tb^3+^ to Eu^3+^ energy transfer. Figure 4g shows negligible rates (<1 s^−1^) that involve high energy gaps (non‐resonant conditions) that will not affect the overall Tb^3+^→Eu^3+^ energy transfer process.

Using these rates, together with the radiative rates (*A_rad_*, Equation S4, Supporting Information) and the multiphonon decay (*W_mp_*, Equation S1, Supporting Information) rates for Eu^3+ 5^D_0_→^7^F_6_ and Tb^3+ 5^D_4_→^7^F_0_ (Table S1, Supporting Information), we constructed the rate equation model (Equations S15–S20, Supporting Information) to estimate the populations of each emitting level (*P*_1_ and *P*_2_) and, consequently, the theoretical *LIR*   =  *P*_1_  /*P*_2_.

The unusually higher intensity of the Eu^3+ 5^D_0_→^7^F_4_ transition compared to ^5^D_0_→^7^F_2_ (Figure 4b) is due to the sensitivity of the Eu^3+^ ion to changes in its local symmetry.^[^ ^100^, ^101^ ^]^ These transitions are governed by the Judd–Ofelt intensity parameters: ^5^D_0_→^7^F_2_ depends only on Ω_2_ while ^5^D_0_→^7^F_4_ depends only on Ω_4_. Each Ω_λ_ reflects the influence of the surrounding ligand field on Eu^3+^ and can be calculated as:

(1) $$\Omega_{\lambda }=\left(2\lambda +1\right)\;\sum_{t,p}\frac{{\left|B_{\lambda ,t,p}\right|}^{2}}{2t+1}$$

where *B*_λ,*t*, *p*_ are odd‐rank ligand field parameters and are determined mainly by the forced electric dipole and Dynamic Coupling (DC) mechanisms in a non‐centrosymmetric system.^[^ ^102^, ^103^ ^]^ Both mechanisms depend on the local symmetry, expressed through spherical harmonics:

(2) $$B_{\lambda ,t,p}\;\propto \;\sum_{i}\frac{Y_{p}^{t*}\left(\theta_{i},\phi_{i}\right)}{R_{i}^{t+1}}$$

where $Y_{p}^{t*}\left(\theta ,\phi \right)$ are complex conjugates of spherical harmonics of rank *t* (odd values, *t *= *λ* − 1 and *λ *+ 1) and component *p* (integer values from −*t* to +*t*). *R_i_* is the distance from Ln^3+^ to the *i*‐th ligating, and *θ* and *ϕ* are the polar and azimuthal angles, respectively.

From these equations, *Ω*_2_ involves lower‐rank ligand field terms (*t* = 1 and 3), whereas *Ω*_4_ involves higher‐rank terms *(t* = 3 and 5). When the local environment of Eu^3+^ approaches a more symmetric geometry (such as the [(Tb/Eu)_9_(acac)_16_(μ_3_‐OH)_8_(μ_4_‐O)(μ_4_‐OH)] complex when encapsulated in silica), contributions from opposite ligands (i.e., the sum in Equation (2)) tend to cancel out. This cancellation effect is significantly stronger on the lower‐rank terms governing *Ω*_2_. Consequently, *Ω*_2_, and thus the ^5^D_0_→^7^F_2_ transition, weakens much faster than *Ω*_4_ (and ^5^D_0_→^7^F_4_), which can be visualized by the simulation in Supporting Video S1 (Supporting Information; through the JOYSpectra web platform),^[^ ^104^ ^]^ showing that the *Ω*_2_ decreased faster than *Ω*_4_ under a C_4v_ to O_h_ symmetry change.

**Figure 5:**
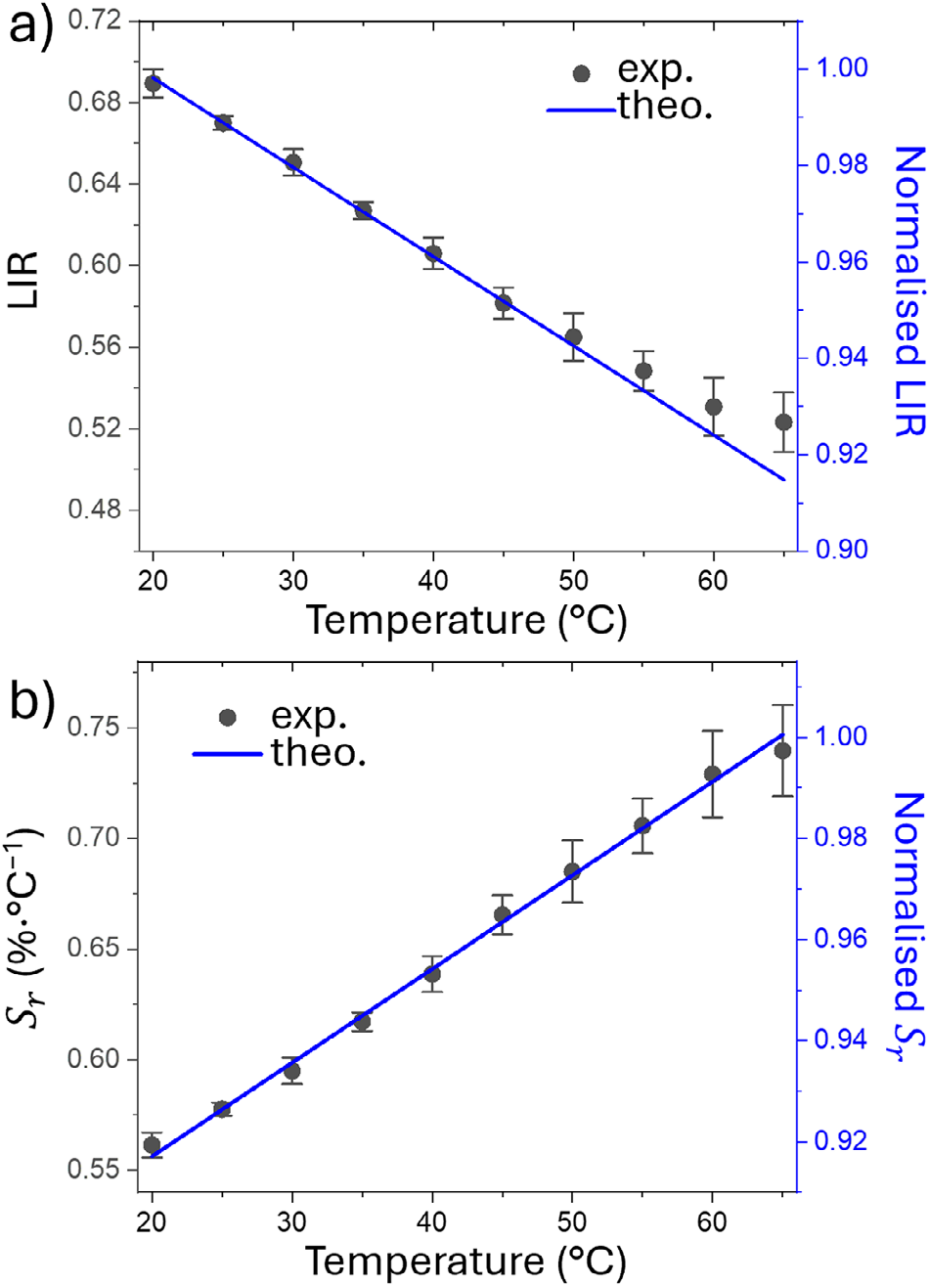
Experimental (black circles) and theoretical (blue lines) thermometric performance of **3A** in water over the 20–65 °C range. a) LIR (^5^D_4_→^7^F_5_/^5^D_0_→^7^F_4_) and b) *S_r_* from 20 to 65 °C temperature range. Error bars correspond to the standard deviation from three consecutive temperature cycles and the theoretical curves are normalized.

The measured LIR as a function of temperature for **3A**, calculated from the integrated emission of the Tb^3+ 5^D_4_→^7^F_5_ (530—560 nm) and the Eu^3+ 5^D_0_→^7^F_4_ (675–715 nm) transitions is demonstrated in **Figure** **5**a. Error bars represent the standard deviation obtained from three consecutive temperature cycles. Over the 20–65 °C range, LIR decreases monotonically with increasing temperature, confirming its suitability as a self‐referencing sensor. Comparable results were observed for sample **3B** (Figure S10b, Supporting Information). The relative thermal sensitivity, *S_r_* (Equation S21, Supporting Information), defined as the percentage change in LIR per degree Celsius,^[^ ^105^ ^]^ is plotted in Figure 5b and Figure S10 (Supporting Information). The maximum *S_r_* reaches 0.75% °C^−1^ at 65 °C for **3A** and 0.70% °C^−1^ at 65 °C for **3B**, values similar to the peak sensitivities (≈1% °C^−1^) typically reported for ratiometric lanthanide thermometers in aqueous media.^[^ ^106^ ^]^ The theoretical *S_r_*, shown as a blue line in Figure 5b for **3A**, is normalised because the rate equation simulations use normalised populations. Nevertheless, the simulated temperature dependencies of both LIR and *S_r_* agree closely with the experimental data. The good relative standard deviation (RSD) indicating measurement repeatability, defined as the maximum RSD among measurement values, is below 5% for **3A** and 11% for **3B**. The measured thermal uncertainty (δT, Equation S23, Supporting Information) is very satisfactory for **3A**, with a value of 1 °C, while it is higher for **3B**, reaching 4 °C.

The absence of Ln^3+^ complex leaching was confirmed by analyzing the luminescence of the remaining solution after **3A** removal. Its emission spectrum, measured strictly in the same conditions, did not present the characteristic Ln^3+^ transitions (Figure S11, Supporting Information).

All luminescent and thermometric measurements in this study were carried out in water at pH = 7.4; comparable emission spectra were also observed at pH = 5.7, while more detailed investigations on aging, stability, and pH‐dependent thermometry, particularly relevant for biomedical applications, will be addressed in future work.

### 2.4 Real‐Time In Situ Temperature Monitoring during Photothermal Heating

The temperature detection by luminescence signal during the photothermal heating was performed with aqueous colloidal solutions of IONP@SiO_2_‐acac/(Tb/Eu)_9_@SiO_2_ **3A,** since these nanoparticles present better *S_r_*. To simultaneously monitor the eigen temperature fluctuation in this colloidal solution during photothermal heating, the Edinburgh FLS1000 spectrofluorimeter was coupled with an 808 nm laser (laser power of 2.58 W cm^−2^), as shown in Figure S12 (Supporting Information). An optical fiber was introduced into the solution to probe the macroscopic temperature (see Supporting Information).

**Figure 6:**
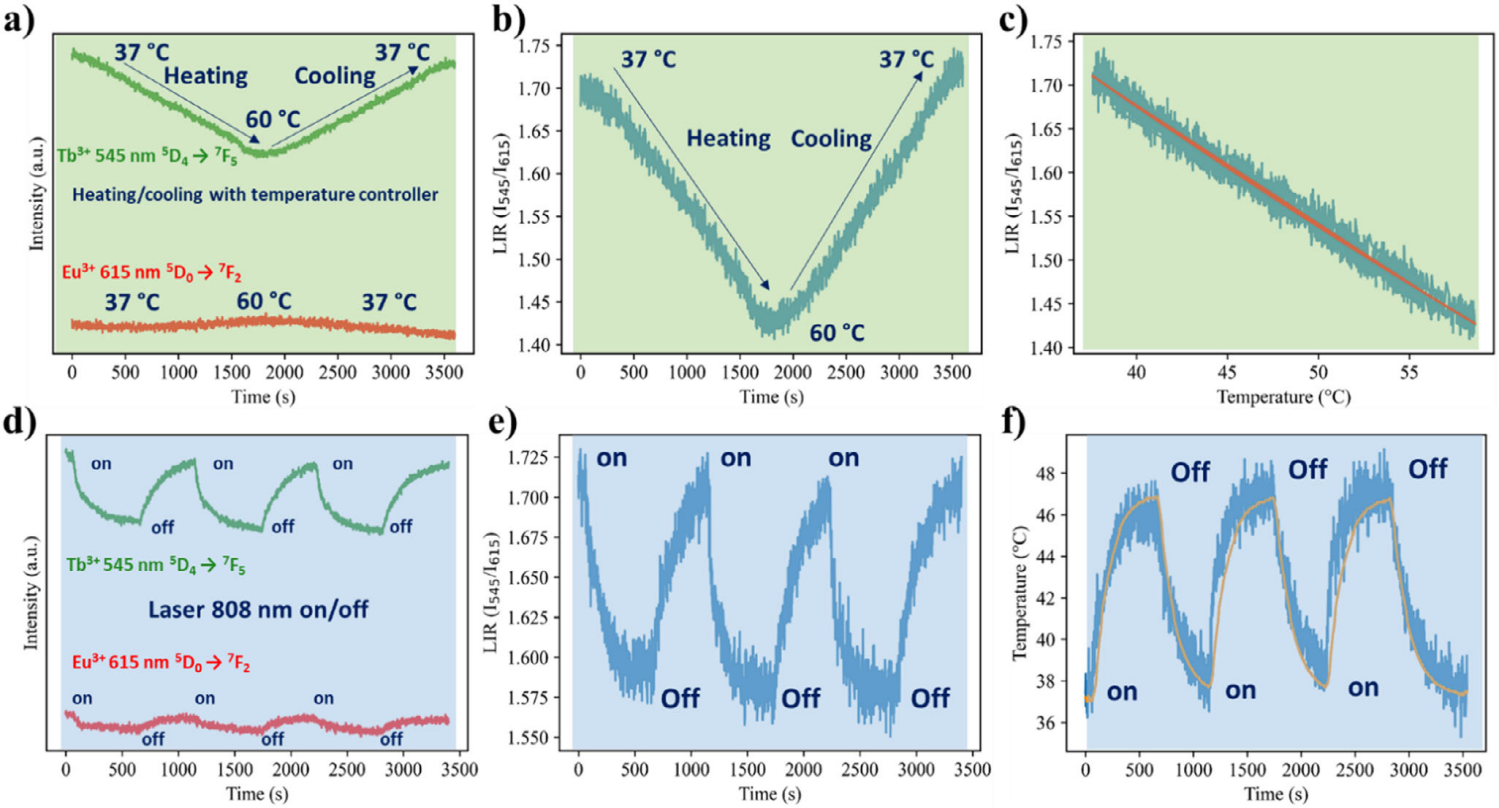
a) Temperature dependences of the emission intensities for ^5^D_4_→^7^F_5_ (Tb^3+^) (green) and ^5^D_0_→^7^F_2_ (Eu^3+^) (orange) transitions of **3A** during the heating up to 60 °C and cooling to 37 °C with thermalization by the temperature controller at 37 °C (the same scale is used for Tb^3+^ and Eu^3+^ related intensities); b) Temperature dependence of LIR (between intensities of ^5^D_4_→^7^F_5_ (Tb^3+^) and ^5^D_0_→^7^F_2_ (Eu^3+^) transitions) during the heating up to 60 °C and cooling to 37 °C regulated by the temperature controller; c) The corresponding calibration curve represented as LIR (I_545/615_) *vs* temperature (blue) and the macroscopic temperature monitored by optical fibre (orange); d) The fluctuation of the emission intensities of ^5^D_4_→^7^F_5_ (Tb^3+^) (green) and ^5^D_0_→^7^F_2_ (Eu^3+^) (red) transitions under irradiation at 808 nm (power 2.58 W cm^−2^) in the on/off mode; e) Fluctuation of corresponding LIR under irradiation in *on/off* mode; f) Temperature variation obtained by using the calibration curve under irradiation at 808 nm in *on/off* mode (blue) and the macroscopic temperature monitored by optical fibre (orange).

First, the local temperature detection during the photothermal heating was performed with the thermalization of the whole colloidal solution at 37 °C. The calibration curves were obtained by using the emission intensities of the main ^5^D_4_→^7^F_5_ (for Tb^3+^) and ^5^D_0_→^7^F_2_ (for Eu^3+^) transitions upon heating/cooling cycles from 37 to 65 °C and back to 37 °C using a thermal controller. **Figure** **6**a shows that the intensity of the Tb^3+ 5^D_4_→^7^F_5_ transition decreases (19%) as the temperature increases, while the Eu^3+ 5^D_0_→^7^F_2_ transition remains almost unaffected. The variation of the LIR between ^5^D_4_→^7^F_5_ (for Tb^3+^) and ^5^D_0_→^7^F_2_ (for Eu^3+^) transitions during the heating from 37 to 65 °C and subsequent cooling from 65 to 37 °C is depicted in Figure 6b. The superposition of the LIR variations during heating/cooling provides the calibration curves (Figure 6c).

Second, the intensity variations for the same ^5^D_4_→^7^F_5_ (for Tb^3+^) and ^5^D_0_→^7^F_2_ (for Eu^3+^) transitions were monitored under 808 nm irradiation (laser power 2.58 W cm^−2^) in *on/off* mode with 10‐minute segments (Figure 6d). As expected, the Tb^3+ 5^D_4_→^7^F_5_ intensity is sensitive to irradiation, decreasing when the laser is *on* and returning to its initial value when the laser is *off*, while the Eu^3+ 5^D_0_→^7^F_2_ intensity does not show significant fluctuation. The corresponding LIR modulation under *on/off* irradiation is shown in Figure 6e. Note that the intensities of the Tb^3+ 5^D_4_→^7^F_5_ and Eu^3+ 5^D_0_→^7^F_2_ transitions for the IONP‐free control SiO_2_‐acac/(Tb/Eu)_9_@SiO_2_ nanoparticles were not sensitive to irradiation, and the corresponding LIR remained constant regardless of whether the laser was *on* or *off* (Figure S13, Supporting Information). Based on the calibration curve depicted in Figure 6c, the temperature dependence relative to irradiation time is illustrated in Figure 6f with blue lines, while the macroscopic temperature of the solution is indicated by the orange line. The heating up to 47 °C and the subsequent cooling back to 37 °C are monitored during each 10‐min segment of the laser *on/off* mode. The temperature measured using our emission probe during photothermal heating aligns perfectly with the macroscopic temperature variation monitored by the optical fiber, indicating the reliability of our nano‐system.

Third, the experiments were conducted in the same conditions but with a colloidal solution thermalized at 20 °C instead of 37 °C (Figure S14, Supporting Information). Our results reveal noticeable thermal inhomogeneity under laser irradiation, with local temperatures recorded by luminescence reaching up to 35 °C, while the bulk solution remained at 31 °C (Figure S14f, Supporting Information). Since the calibration curve was well‐validated, these discrepancies suggest nanoscale thermal gradients, likely due to reduced convection, decreased Brownian motion, localized heat accumulation, and temperature‐dependent heat dissipation.^[^ ^46^ ^]^ These findings imply that macroscopic measurements may underestimate local temperatures under certain conditions, highlighting the need for further research into nanoscale heat transfer dynamics. This promising result opens the door for further exploration of the “thermal inhomogeneity” effect under photothermal heating, with future research aimed at optimizing the system's effectiveness in various conditions.

## 3 Conclusion

In summary, this study introduces a new multifunctional system for real‐time in situ temperature monitoring at the nanoscale *via* ratiometric luminescence thermometry during photothermal heating. The system features challenging, well‐defined heater/thermometer nano‐objects, consisting of IONP core stellate‐like silica shell nanoparticles, with pores initially loaded and later sealed to trap and protect the luminescent complex [(Tb/Eu)_9_(acac)_16_(μ_3_‐OH)_8_(μ_4_‐O)(μ_4_‐OH)] inside. These nanoobjects were synthesized by a multistep approach involving the synthesis of IONP@SiO_2_ shell nanoparticles functionalized with an acetylacetonate moiety, followed by loading with the complex and clogging the pores with silica to protect the complex and prevent its leaching. The obtained multifunctional IONP@SiO_2_‐acac/(Tb/Eu)_9_@SiO_2_ nanoparticles exhibit a size of ≈100 nm and retain their morphology after complex loading and pore sealing. Structurally well‐defined, these nanoparticles consist of a single IONP core encased within a precisely engineered stellate‐like silica shell, available with either smaller or larger pore openings depending on the pH.

Solid‐state photoluminescence demonstrated the successful loading of [(Tb/Eu)_9_(acac)_16_(μ_3_‐OH)_8_(μ_4_‐O)(μ_4_‐OH)] into the silica shells. Moreover, while the excitation and emission transitions exhibit similar positions before and after loading the complex, variations in the intensity ratio between specific transitions are evident. They have been attributed to a slight alteration in the complex's geometry during the encapsulation, involving a shortening of intramolecular Ln^3+^‐ Ln^3+^ distances, providing the Tb^3+^ to Eu^3+^ energy transfer enhancement. The theoretical modeling demonstrated an exceptionally high Tb^3+^→Eu^3+^ energy transfer rate, which may reach on the order of 10^11^ s^−1^. The energy transfer dynamics and thermometric performance were also simulated through state‐of‐the‐art models in the literature.

The macroscopic heating of IONP@SiO_2_‐acac/(Tb/Eu)_9_@SiO_2_ nanoparticles in aqueous colloidal solutions was investigated under 808 nm light irradiation and demonstrated expected concentration‐dependent macroscopic heat. This latter is comparable with the macroscopic temperature elevation obtained for IONP nanoparticles without silica shell measured in the same conditions, suggesting that the open porosity of the stellate‐like silica shell is favorable for rapid water exchange and heat dissipation. Therefore, the stellate‐like silica shell serves additional roles such as enhancing colloidal stability, providing functionality for luminescent complex insertion, while maintaining good photothermal response.

The temperature dependence of the photoluminescence realized in aqueous colloidal solutions in the 20–65 °C range indicates that nanoparticles can be used as efficient nanothermometers with good cyclability and a maximum relative sensitivity of 0.75% °C^−1^ at 65 °C, enabling precise temperature detection compared to other emissive temperature probes. Employing the Tb^3+^ to Eu^3+^ energy transfer driven thermometer, we successfully modeled the Tb^3+^→Eu^3+^ energy transfer and demonstrated real‐time in situ temperature monitoring during photothermal heating under continuous 808 nm irradiation. These latter revealed noticeable nanoscale thermal gradients between the nanoparticles' surface and environment, depending on thermal conditions. These findings highlight the importance of considering local temperature variations, as macroscopic measurements may systematically underestimate the true local temperatures and open the door for further exploration into the mechanisms of thermal inhomogeneity.

The authors have cited additional references within the Supporting Information.^[^ ^39^, ^82^, ^84^, ^85^, ^87^, ^89^, ^97^, ^107^, ^108^, ^109^, ^110^, ^111^, ^112^, ^113^, ^114^, ^115^, ^116^, ^117^, ^118^, ^119^, ^120^, ^121^, ^122^, ^123^, ^124^, ^125^ ^]^

## Conflict of Interest

The authors declare no conflict of interest.

## Supporting information



Supporting Information

Video

## Data Availability

The data that support the findings of this study are available in the supplementary material of this article.
