# Supplementary material for: Photothermal Heating and Real‐Time In Situ Luminescent Thermometry with Iron Oxide Core‐Silica Shell Nano‐Objects
Source: Small. 2025 Oct 22;21(49):e08497. doi: 10.1002/smll.202508497 (PMC12696789; doi:10.1002/smll.202508497)
Supplement: Supplementary file 1 — Supporting Information [file SMLL-21-e08497-s002.docx]

**Photothermal Heating and Real-Time *In-Situ* Luminescent Thermometry with Iron Oxide Core-Silica Shell**

**Nano-objects**

Farah Abdel Sater,^[a]^ Gautier Félix,^[a]^ Saad Sene,^[a]^ Udara Bimendra Gunatilake,^[a]^ Basile Bouvet,^[a]^ Tristan Pelluau,^[a]^ Erwan Oliviero,^[a]^ Albano N. Carneiro Neto,^[b,c]^ Luís D. Carlos,^[b]^ Belén Albela,^[d]^ Laurent Bonneviot,^[d]^ Yannick Guari*^[a]^ and Joulia Larionova*^[a]^

*[a] F. Abdel Sater, B. Bouvet, Dr. S. Sene, Dr. G. Félix, Dr. T. Pelluau, Dr. Erwan Oliviero, Dr. Y. Guari and Pr. J. Larionova* *ICGM, Univ. Montpellier, CNRS, ENSCM, Montpellier, France.* *E-mail:* *[joulia.larionova@umontpellier.fr](mailto:joulia.larionova@umontpellier.fr), yannick.guari@umontpellier.fr*

*[b]* *Dr. A. N. Carneiro Neto, L. D. Carlos,* *Department of Physics and CICECO – Aveiro Institute of Materials, University of Aveiro, 3810-193, Aveiro, Portugal.*

*[c] Dr. A. N. Carneiro Neto, Max-Planck-Institut für Kohlenforschung, Kaiser-Wilhelm-Platz 1, D-45470 Mülheim an der Ruhr, Germany.*

*[d]* *Dr. Belén Albela, Pr. L. Bonneviot,* *Laboratoire de Chimie, ENS de Lyon, Université de Lyon, Lyon, France.*

**Contents**

[EXPERIMENTAL PART 4](#_Toc210054776)

[1. General Procedures 4](#_Toc210054777)

[1.1. Syntheses and Characterization 4](#_Toc210054778)

[1.2. Complexes’ leaching experiments: 6](#_Toc210054779)

[2. Methods 6](#_Toc210054780)

[2.1. Photoluminescence and thermometry measurements 6](#_Toc210054781)

[2.2. Photothermia experiments 7](#_Toc210054782)

[2.3. Temperature detection during photothermal heating 7](#_Toc210054783)

[2.4. Photothermal conversion efficiency 7](#_Toc210054784)

[FIGURES 9](#_Toc210054785)

[**Figure S1**. Infrared spectra of: a) samples **1A** – **3A** presenting stellate-like morphology of the silica shell, and b) **1B** – **3B** having stellate-like morphology of the silica shell with more open porosity. 9](#_Toc210054786)

[**Figure S3**. Size distributions for: a) IONP@SiO_2_-acac **1A**; b) IONP@SiO_2_-acac **1B**; c) IONP@SiO_2_-acac/(Tb/Eu)_9_ **2A**; d) IONP@SiO_2_-acac/(Tb/Eu)_9_ **2B**; c) IONP@SiO_2_-acac/(Tb/Eu)_9_@SiO_2_ **3A**; c) IONP@SiO_2_-acac/(Tb/Eu)_9_@SiO_2_ **3B**. The red line represents the Gaussian fit. 10](#_Toc210054787)

[**Figure S4.** Nitrogen adsorption isotherms of **1A** (black) and **3A** (red). 11](#_Toc210054788)

[**Figure S5**. Hydrodynamic diameter distribution for **1A** (a), **2A** (b), **3A** (c), **1B** (d), **2B** (e) and **3B** (f); g) Comparison of hydrodynamic distributions of sample **3A** freshly prepared and after 27 days of storage in water. 12](#_Toc210054789)

[**Figure S6**. a) T vs time curves performed for aqueous colloidal solutions of **3A** (a) and **3B** (b) with 9.1 mg·mL^−1^ of IONP performed under irradiation at 808 nm (2.5 W·cm^−2^). The red curves represent the theoretical fit that was employed to extract the photothermal conversion efficiency. The solving process is delineated in section 2.4 of the ESI. 13](#_Toc210054790)

[**Figure S7**. a) ΔT vs time curves performed for aqueous colloidal solutions of IONPs coated with gallic acid with different concentrations of Fe_3_O_4_ performed under irradiation at 808 nm (2.5 W cm^-2^) with ΔT representing the difference between the temperatures of colloidal solutions and water as a reference; b) Corresponding variation of ΔT vs IONPs concentration. 14](#_Toc210054791)

[**Figure S8**. a) Room temperature excitation spectra of IONP@SiO_2_-acac/(Tb/Eu)_9_@SiO_2_ **3B** monitored at λ_em_ = 545 nm (green), 615 nm (red) and 700 nm (orange) in water; b) Room temperature emission spectra of IONP@SiO_2_-acac/(Tb/Eu)_9_@SiO_2_ **3B** under excitation at 315 nm recorded from 450 to 720 nm in water. * Raman scattering peak of water. 15](#_Toc210054792)

[**Figure S9**. a) Excitation spectra of [(Tb/Eu)_9_(acac)_16_(μ_3_-OH)_8_(μ_4_-O)(μ_4_-OH)]·H_2_O complex monitored at λ_em_ = 545 (green) and 615 nm (red) measured at room temperature in solid state, b) Emission spectra of [(Tb/Eu)_9_(acac)_16_(μ_3_-OH)_8_(μ_4_-O)(μ_4_-OH)] H_2_O complex performed with λ_ex_ = 328 nm at room temperature in solid state. 15](#_Toc210054793)

[**Figure S10**. a) Emission spectra of **3B** (λ_ex_ = 315 nm) recorded in water at temperatures from 20 to 65 °C, showing the progressive decrease of Tb^3+^ intensities. b) Luminescence intensity ratio (LIR = I_545_/I_700_) between the Tb^3+^ ^5^D_4_→^7^F_5_ and Eu^3+^ ^5^D_0_→^7^F_4_ transitions plotted against temperature for **3B**. The solid line is a guide to the eye; error bars denote the standard error of the mean from three consecutive temperature cycles. 16](#_Toc210054794)

[**Figure S11**. Room temperature emission spectra under excitation at 315 nm in water for IONP@SiO_2_-acac/(Tb/Eu)_9_@SiO_2_ **3A** (black) and for the supernatant solution of **3A** remaining after heating at 60 °C (red) and removal of the nanoparticles performed in the same conditions. 16](#_Toc210054795)

[**Figure S12.** Schematic representation (a) and a photograph (b) of the setup for the luminescence monitoring during the photothermal heating. 17](#_Toc210054796)

[**Figure S13**. a) Emission peaks at 545 and 615 nm of SiO_2_-acac/(Tb/Eu)_9_@SiO_2_ nanoparticles (λ_ex_ = 300 nm) recorded as a function of time with temperature fixed at 37 °C; b) LIR (between the ^5^D_4_→^7^F_5_ (Tb^3+^) and ^5^D_0_→^7^F_2_ (Eu^3+^) transitions vs time for SiO_2_-acac/(Tb/Eu)_9_@SiO_2_ nanoparticles. 17](#_Toc210054797)

[**Figure S14.** a) Temperature dependences of the emission intensities for ^5^D_4_→^7^F_5_ (Tb^3+^) (green) and ^5^D_0_→^7^F_2_ (Eu^3+^) (orange) transitions during the heating up to 50 °C and cooling of the sample **3A** to 20 °C regulated by the temperature controller with the thermalisation at 20 °C (the same scale is used for Tb^3+^ and Eu^3+^ related intensities); b) Temperature dependence of LIR (between intensities of ^5^D_4_→^7^F_5_ (Tb^3+^) and ^5^D_0_→^7^F_2_ (Eu^3+^) transitions) during the heating up to 50 °C and cooling of the sample to 20 °C regulated by the temperature controller at 20 °C; c) The corresponding calibration curve represented as LIR (I_545_/I_615_) vs temperature (blue) and the macroscopic temperature monitored by optical fibre (orange); d) The fluctuation of the emission intensities of ^5^D_4_→^7^F_5_ (Tb^3+^) (green) and ^5^D_0_→^7^F_2_ (Eu^3+^) (red) transitions under light irradiation at 808 nm (power 2.5 W·cm^−2^) in the on/off mode; e) Fluctuation of LIR (between intensities of ^5^D_4_→^7^F_5_ (Tb^3+^) and ^5^D_0_→^7^F_2_ (Eu^3+^) transitions) under light irradiation at 808 nm in the on/off mode (power 2.5 W·cm^−2^); f) Temperature variation obtained by using the calibration curve under light irradiation at 808 nm in the on/off mode (power 2.58 W·cm^−2^) (blue) and the macroscopic temperature monitored by optical fibre (orange). 18](#_Toc210054798)

[THEORETICAL MODELLING 19](#_Toc210054799)

[1. Multiphonon, radiative rates, and lifetimes 19](#_Toc210054800)

[**Figure S15.** Molecular structure of the {Ln_9_} cluster, highlighting the ten O–H groups that coordinate the nine lanthanide ions. Oxygen atoms are shown in red, lanthanide centers in greenish-blue, and hydrogen atoms of the coordinating hydroxyls in white, illustrating how the ten hydroxide ligands may interact with Ln^3+^. 20](#_Toc210054801)

[**Table S1**. Radiative and multiphonon parameters to obtain the lifetime $\tau$. 21](#_Toc210054802)

[2. Pairwise Tb-to-Eu energy transfer rates 21](#_Toc210054803)

[3. Effective Tb-to-Eu energy transfer rates 23](#_Toc210054804)

[**Figure S16.** Expanded supercell representation showing only the {Ln_9_} clusters. The distances between each lanthanide ion within a single {Ln_9_} cluster (e.g., 3.439 Å, 3.440 Å, 3.517 Å, and 3.548 Å) are illustrated. 24](#_Toc210054805)

[**Table S2**. Simulated coefficients of occurrences for a Eu^3+^/Tb^3+^ ratio of 1:9 for the five shortest Tb^3+^-Eu^3+^ distances. 24](#_Toc210054806)

[4. Rate equations modelling and thermometric simulation 24](#_Toc210054807)

[5. Thermometric performance 25](#_Toc210054808)

[REFERENCES 26](#_Toc210054809)

# EXPERIMENTAL PART

# General Procedures

TEOS was purchased from abcr (Karlsruhe, Germany), hexadecyltrimethylammonium ptoluenesulfonate (CTATos) and ethanol were purchased from Merck (Darmstadt, Germany), Ferric hydroxide oxide (FeO(OH) hydrated, 30-50 mesh), oleic acid (90%, OAm), oleylamine (90%, OL), ammonium nitrate, triethanolamine (TEAH_3_) were purchased from Sigma-Aldrich (Steinheim, Germany), n-docosane (99%) was purchased from Acros organic. The 3-[3- (Triethoxysilyl)propyl]pentane-2,4-dione)] (acac-Si) was synthesized according to the previously published method.^[1]^ The synthesis of [(Tb/Eu)_9_(acac)_16_(μ_3_-OH)_8_(μ_4_-O)(μ_4_-OH)]·H_2_O complex (Tb^3+^/Eu^3+^ = 9/1) was performed following the previously published method. ^[2,3]^

## 1.1. Syntheses and Characterization

**Synthesis of pristine IONP stabilized by oleate and oleyl amine***:* Pristine IONP of *ca*. 26 nm stabilized by oleate (OA) and by oleyl amine (OAm) were prepared by adapting a previously published thermal decomposition method (at 350 °C) by using FeO(OH) as the iron precursor in n-docosane as a solvent.^[4]^ First, a flask containing a mixture of FeO(OH) (2.1 mmol, 0.186 g), oleic acid (10 mmol, 3.17 g), and n-docosane (5.02 g) was connected to a Schlenk line to remove moisture and oxygen for 30 min at room temperature under vacuum and magnetic stirring. Subsequently, the flask was heated to 350 °C under argon flow with a heating rate of 10 °C·min^-1^. The solution was maintained at 350 °C for a further 90 min under stirring and argon flow. The heating was turned off, and the mixture was cooled down to 200 °C. When the temperature reached this value, the system was opened to air, and the temperature was maintained at 180 °C for a further 90 min to realize oxidation of the nanoparticles (from FeO to Fe_3_O_4_). After this period, the heating source was removed. When the temperature of the solution reached 50 °C, cyclohexane (15 mL) was added in order to precipitate the nanoparticles. The obtained nanoparticles were washed twice by dispersing in diethyl ether, followed by precipitation with ethanol (1:1 v/v), and then recovered using centrifugation (20,000 rpm, 10 min). Oleylamine (200 µL) is added to the collected material as an additional stabilizer. The resultant oleate/oleylamine-capped IONP/OA/OAm nanoparticles were finally dispersed in chloroform (15 mL) for further use.^[5]^

**Synthesis of functionalized core@shell IONP@SiO_2_-acac nanoparticles with different stellate-like shell morphology (1A) and (1B)**: The synthesis of nano-objects containing the IONP core enwrapped by mesoporous silica shell functionalized with acetylacetone (acac) derivative was adapted from the work of A. Adam et al.^[6]^ In a typical procedure, 48 milligrams of CTATos and either 8.7 or 6 milligrams of TEAH₃ were dissolved in 5 millilitres of water at 80 °C for 1 hour, resulting in pH values of 7 and 5.6, respectively. Then, the IONP/OA/OAm (1 mL, 10 mg) was added dropwise using a syringe under vigorous stirring (500 rpm), and the solution was heated to 65 °C to evaporate the chloroform for 1 h until a black coloration appeared. The sol-gel reaction started after the addition of TEOS (0.36 mL, 0.4 mL) while the temperature was increased from 65 to 80 °C. The mixture was then stirred for 60 min. Afterward, 0.04 mL TEOS and 40 µL of 3-[3-(Triethoxysilyl)propyl]pentane-2,4-dione)] (acac-Si) were added at 80 °C, and the mixture was stirred for an additional 2h, giving sample **1A** with a stellate-like silica shell morphology. For sample **1B** having more pronounced opening of the radial porosity, a direct injection of 40 µL of the acetylacetonate derivative (acac-Si) with the 0.4 mL TEOS was performed, and the mixture was stirred for 2h at 80 °C. Note that at lower pH levels, the competition advantages the shift towards the tosylate anion, leading to conditions of "weak templating" favouring, therefore, the formation of the more open stellate morphology. Three washings of 30 min each were performed with an ethanolic solution of ammonium nitrate at 480 mol·L^-1^ under sonication and vortex for the extraction of the surfactant CTATos. Solvent extraction was chosen in order to preserve the acac functionality and to prevent further nanoparticle aggregation.^[2,7,8]^ The nanoparticles were then washed with ethanol and finally dispersed in EtOH (10 mL) and stored at room temperature until further use.^[2]^

**Characterization of 1A**: IR (KBr): δ(Si−O−Si)= 470 cm^−1^ (SiO_2_), ν(Si−O−Si)= 800-1094 cm^−1^ (SiO_2_), ν(Si-OH)=957 cm^−1^, ν(C=C)= 1350-1400 cm^−1^ (acac-Si) ,ν(C=O)=1725, 1690 and 1632 cm^−1^, ν(C=O)= 1700 cm^−1^ (acac-Si), ν(C-H)= 2900-3000 cm^−1^ (acac-Si), ν(Fe-O)= 570 cm^−1^. d_TEM_= 103.7 ± 4.7 nm. EDS: Si/Fe= 91/9

***Characterization of 1B***: IR (KBr): δ(Si−O−Si)= 464 cm^−1^ (SiO_2_), ν(Si−O−Si)= 795-1090 cm^−1^ (SiO_2_), ν(Si-OH)=960 cm^−1^, ν(C=C)= 1350-1400 cm^−1^ (acac-Si),ν(C=O)=1720, 1690 and 1635 cm^−1^, ν(C=O)= 1700 cm^−1^ (acac-Si), ν(C-H)= 2900-3000 cm^−1^ (*acac*-Si), ν(Fe-O)= 570 cm^−1^. d_TEM_= 93.1 ± 4.6 nm. EDS: Si/Fe= 90/10

**Synthesis of IONP@SiO_2_-acac/(Tb/Eu)_9_ with stellate-like morphology (3A) and (3B)***:* First, the encapsulation of the [(Tb/Eu)_9_(acac)_16_(μ^3^-OH)_8_(μ^4^-O)(μ^4^-OH)] (Tb^3+^/Eu^3+^ = 9/1) compound (denoted here as (Tb/Eu)_9_) was prepared by adapting the previously published procedure by adding 4.6 mg of the complex (in 5 mL methanol) to the IONP@SiO_2_-acac nanoparticles **1A** or **1B** (10 mg) dispersed (in 5 mL of MeOH). The mixture was stirred at 65 °C under reflux for 2h. The (Tb/Eu)_9_ complex-containing nanoparticles, IONP@SiO_2_-acac/(Tb/Eu)_9_ (**2A**) or (**2b**), were washed two times with water (20,000 rpm, 10 min) and finally dispersed in ultra-pure water (6 mL). In order to avoid the complex’s leaching, the clogging of the silica porosity was performed by dispersing the obtained **2A** or **2B** in 6 mL of ultra-pure water and adding 80 µL of TEOS at 80 °C. The mixture was stirred at 80 °C overnight. The collected IONP@SiO_2_-acac/(Tb/Eu)_9_@SiO_2_ nano-objects (**3A**) and (**3B**) were then washed three times with water (20,000 rpm, 10 min) and finally dispersed in ultra-pure water (10 mL) for storage.

EDS (Fe, Si, Tb, Eu) and ICP (Fe, Tb) analyses indicated that samples **2A** and **2B** achieved loadings of 0.0041 and 0.0227 units of complex per SiO_2_ unit (0.037 and 0.205 of Tb/Eu units per unit of SiO_2_), respectively.

**Characterization of 3A**: IR (KBr): δ(Si−O−Si)= 460 cm^−1^ (SiO_2_), ν(Si−O−Si)= 800-1094 cm^−1^ (SiO_2_), ν(C=C)= 1350-1400 cm^−1^ (acac-Si), ν(C=O)= 1510 cm^−1^, ν(C=O)= 1696-1630 cm^−1^ (acac-Si), ν(C-H)= 2850-2928 cm^−1^ (acac-Si), ν(Fe-O)= 566 cm^−1^ . d_TEM_= 109.8 ± 4.3 nm.

EDS: Si/Tb = 47.6/1; Tb/Eu = 9.5; ICP: Tb/Eu = 20.8.

*Characterization of 3b*: IR (KBr): δ(Si−O−Si)= 470 cm^−1^ (SiO_2_), ν(Si−O−Si)= 800-1095 cm^−1^ (SiO_2_), ν(C=C)= 1350-1400 cm^−1^ (acac-Si), ν(C=O)=1770, 1690 and 1635 cm^−1^, ν(C=O)= 1700 cm^−1^ (acac-Si), ν(C-H)= 2900-3000 cm^−1^ (acac-Si), , ν(Fe-O)= 560 cm^−1^. d_TEM_= 107.3 ± 5.2 nm.

EDS: Si/Tb = 13.4/1; Tb/Eu = 9.9; ICP : Tb/Eu = 9.1/1.

## *1.2. Complexes’ leaching experiments*:

The nanoparticles **3A** or **3B** (~ 3 mg) were dispersed in water and then heated at 80°C for several hours under stirring. Then, they were removed by centrifugation and the photoluminescence spectra were recorded for the remaining solutions. The excitation and emission spectra were flat and did not show the characteristic bands of complexes, proving that the complexes remain confined in the silica nanoparticles.

# Methods

Transmission electron microscopy (TEM) images were recorded at 100 kV (JEOL 1400 Flash). Samples for TEM measurements were deposited from suspensions on copper grids covered with carbon film and allowed to dry before observation. The size distribution histograms were determined using enlarged TEM micrographs taken at a magnification of 100 K on a statistical sample of ca. 100 nanoparticles. SEM/EDS microscopy was performed on an FEI Quanta FEG 200 instrument. The powders were deposited on an adhesive carbon film and analysed under high vacuum. The quantification of heavy elements was carried out using Oxford Instruments AZTEC software, with a dwell time of 3 µs. HRTEM and STEM-EDS measurements were performed on a JEOL 2200 FS microscope operated at 200 kV and an Oxford Instruments SDD EDX detector XMaxN 100 TLE (100 mm2 – windowless). Hydrodynamic diameter measurements were performed on Malvern nanoseries, Zetasizer NanoZS (Model ZEN3600) in a DTS1070C Zetacell (for the zeta potential) in water at 25 °C with an equilibration time of 120 s with automatic measurement, and the data were treated with Zetasizer software using a Smoluchowski model. ICP-AES analysis was performed by using a Spectro Arcos ICP (AMETEK Materials Analysis). The samples were digested in 10 M NaOH before being diluted to obtain 10 mL of a final solution in 1% HNO_3_. Infrared (IR) spectra were recorded using attenuated total reflectance as KBr disks (1 wt% of sample) or powders on a PerkinElmer Spectrum Two FT-IR spectrophotometer with four acquisitions at a resolution of 4 cm^−1^. Nitrogen adsorption and desorption isotherms at 77 K were measured using a TriStar 3000 (V6.06 A). Prior to the sorption experiment, the samples were dried under vacuum at 80 °C for 12 h.

## 2.1. Photoluminescence and thermometry measurements

Emission and excitation spectra were measured at room temperature (20 °C) in water, using a spectrofluorometer Edinburgh FLS1000. The excitation source was a 450 W Xe arc lamp. The spectra were corrected for the detection and optical spectral response of the spectrofluorometer. Photoluminescent measurements as a function of temperature (thermometry) were performed by using the temperature setup incorporated into the Edinburgh spectrofluorometer. Measurements in water were performed using solutions of 4 mg·mL^−1^ nanoparticles. Emission spectra were recorded in the temperature range from 20 to 65 °C. At each temperature step, a period of 3 min was given to allow the temperature to stabilize, and then 1 emission spectrum was recorded with a dwell time of 0.2 s and a step of 1 nm.

## 2.2. Photothermia experiments

The photothermal efficiency of the compounds studied was measured by placing 300 µL of a solution containing 659 µg of Fe dispersed in water in a quartz NMR tube placed in a quartz cuvette. The cuvette was then placed in line with the laser, which irradiated at 808 nm with a power of 2.58 W·cm^−2^. Finally, a fibre optic was placed in the solution, enabling the temperature reading every 30 seconds.

## 2.3. Temperature detection during photothermal heating

A real-time measurement was conducted on **3A** in water with a concentration of 20 mg·mL^−1^. An 808 nm laser with a power of 2.58 W·cm^−2^ was coupled with the Edinburgh FLS1000 spectrofluorometer. A total of 200 µL of the sample was placed in a quartz NMR tube, which was subsequently placed in a quartz cuvette within the spectrofluorometer. The sample was aligned with the light beam so that the beam and the laser hit the same spot. Additionally, the fibre optic was inserted into the sample in a manner that did not contact the laser beam. The temperature was fixed at 37 or 20 °C using a Peltier device. Subsequently, a multikinetic scan was registered, with λ_exc_ set to 300 nm, and the emission peaks at 545 nm and 615 nm were recorded for 10 minutes. The laser was then activated at maximum power for 10 minutes, after which it was deactivated for 10 minutes. This cycle was repeated three times. Afterward, the Peltier device was set at 60 or 50 °C with a temperature ramp of 2°C per minute. The temperature rise was recorded, followed by a return to 37 or 20 °C. The recorded temperature data will later be used to create a calibration curve for future reference. It should be noted that all temperature measurements were conducted using a fibre optic sensor. Each experiment was performed 3 times in order to verify the reproducibility of the measurements.

## 2.4. Photothermal conversion efficiency

The photothermal conversion efficiency (η) was extracted using a model that accounts for heat diffusion and natural convection of the air. The model was solved using the COMSOL software. ^[9]^ A Python routine utilizing the MPh library was employed for curve optimization *via* a non-linear least squares method. The model takes into account the light absorption of the media at a wavelength of 808 nm. It accurately replicates the experimental conditions, including light irradiation, the volume of the irradiated area, the shape of the sample, and the material of the container. The thermal flux term between the glass container and the surrounding environment was optimized during the fitting process. A volume of air was introduced above the liquid volume of the sample. In both the irradiated liquid sample and the air volume, natural air convection was calculated and incorporated into the heat equation. To take into account the heat production due to the laser, a thermal source term was added to the equation. It is important to note that this term takes into account two factors: firstly, the exponential loss of light with distance due to the absorption of the laser beam within the sample, and secondly, the radiation-to-heat conversion ratio. The source term is written as:

| $P\left( d \right)=P_{LASER}\cdot\alpha\cdot\exp\left( -\alpha\cdot d \right)\cdot\eta$ |  |
| --- | --- |

with *P(d)* the power density in W/cm^3^, *P_LASER_* the power of the laser in W/cm^2^, *α* the absorption coefficient in cm^-1^ at 808 nm, *d* the distance from the incident point of the laser to the distance *d* in cm, and *η* the radiation-to-heat conversion ratio.

# FIGURES


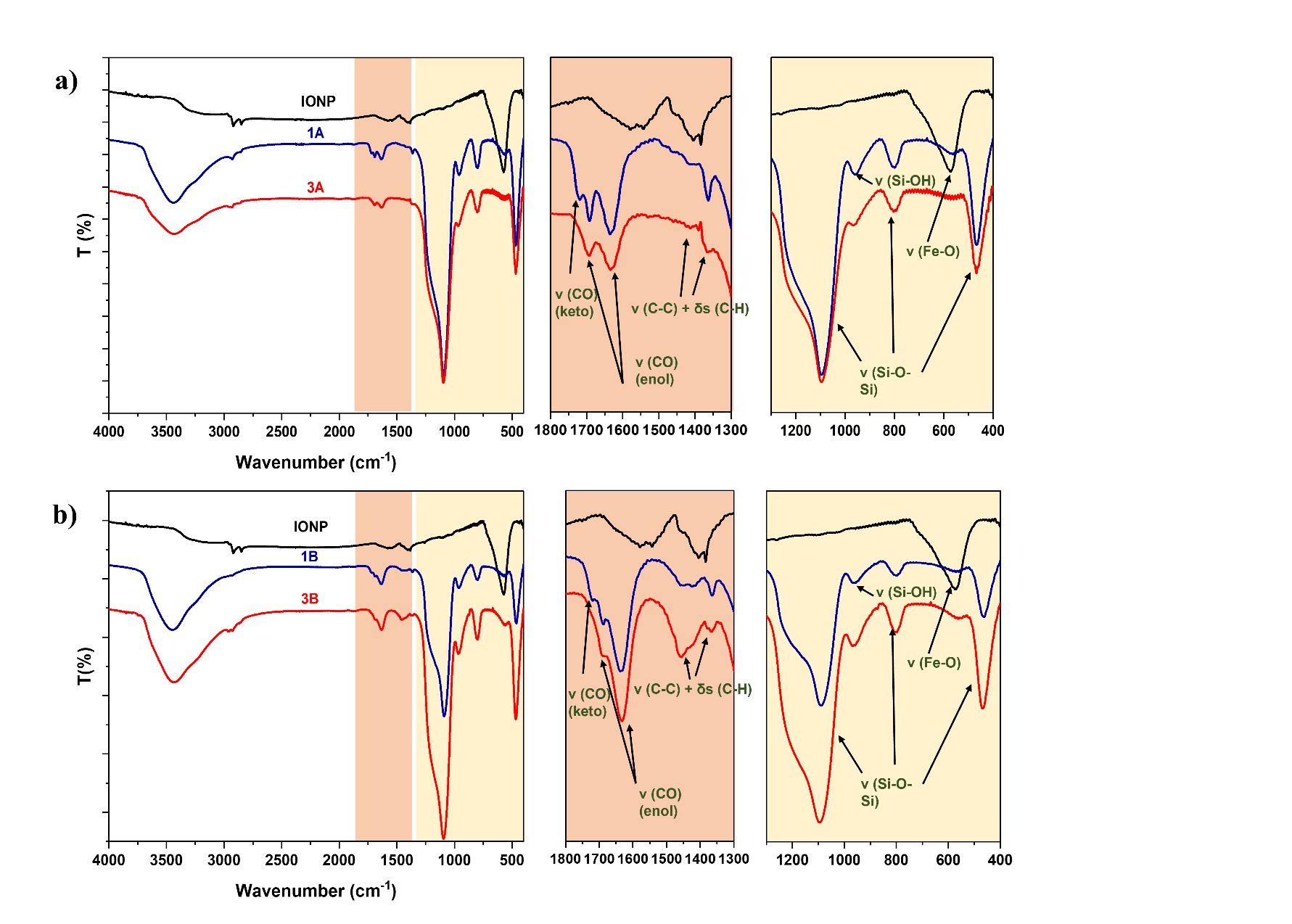


## Figure S1. Infrared spectra of: a) samples **1A** – **3A** presenting stellate-like morphology of the silica shell, and b) **1B** – **3B** having stellate-like morphology of the silica shell with more open porosity.

The IR spectra of **1A** and **1B** present characteristic silica vibrations (ν(Si-O-Si) at 1094 and 800 cm^−1^, δ(Si-O-Si) at 470 cm^−1^, ν(Si-OH) at 957 cm^−1^), as along with bands attributed to the grafted acac function (ν(C=O) at 1725, 1690, and 1632 cm^−1^ in the keto and enol forms, ν(C=C) at 1350-1400 cm^−1^) as well as the C-H vibrational bands between 2900-3000 cm^−1^. Moreover, they show ν(Fe-O) vibration at 570 cm^−1^ of the IONP core. IR spectra for **3A** and **3B**, containing the incorporated luminescent compound, show the disappearance of ν(C=O) vibrations from the free acac moiety at 1716 cm^−1^, as observed in **1A** and **1B**, attesting to the presence of the complex inside the pores.


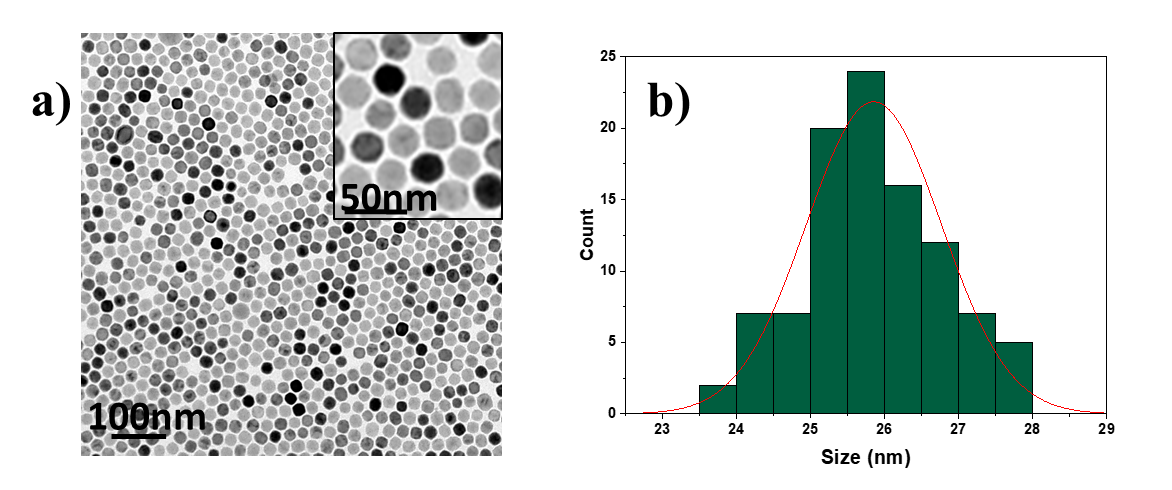
**Figure S2.** Bright field TEM image (a) and size distribution (b) of the pristine IONP/OA/OAm nanoparticles. The red line represents the Gaussian fit.


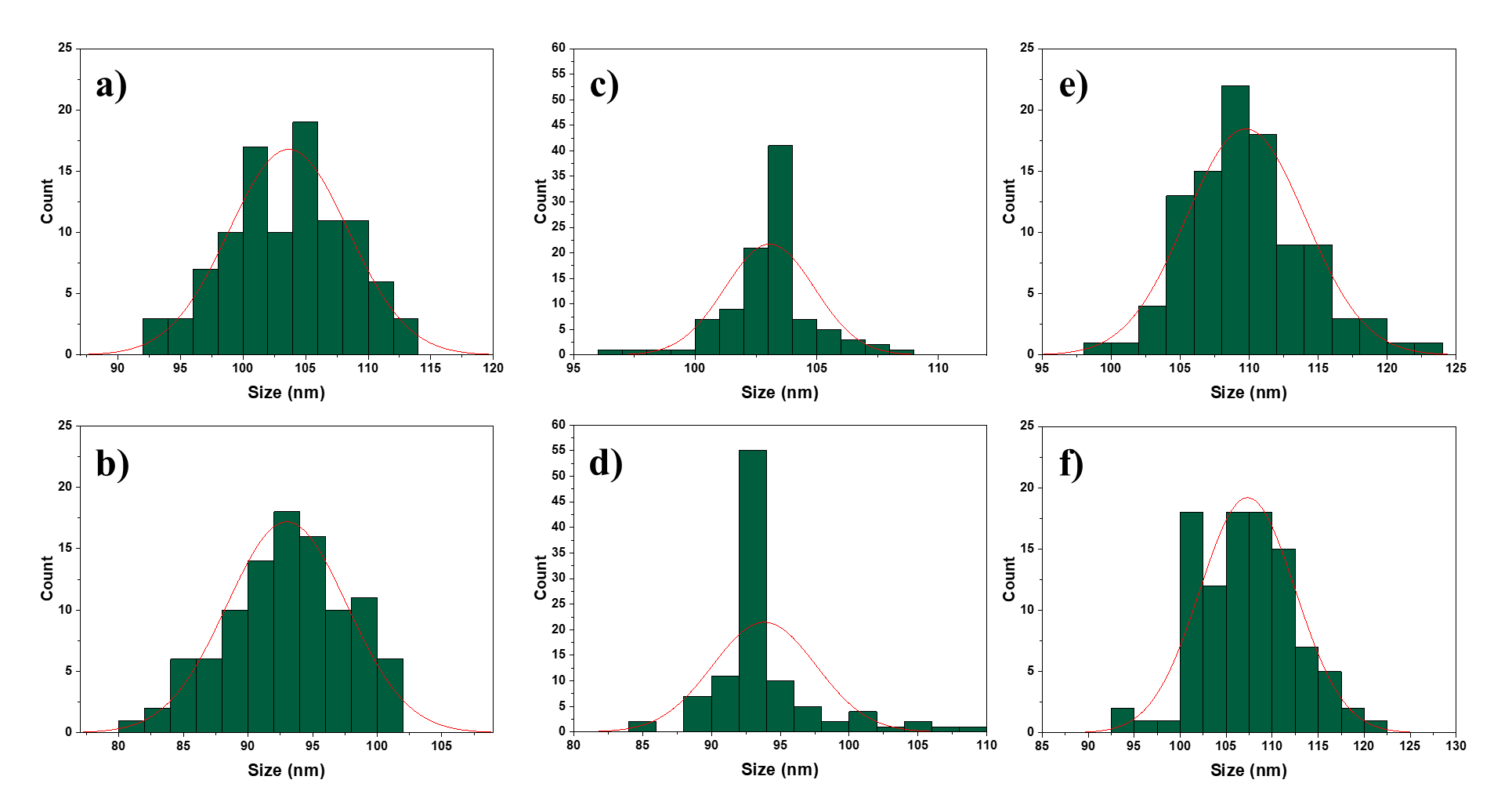


## Figure S3. Size distributions for: a) IONP@SiO_2_-acac **1A**; b) IONP@SiO_2_-acac **1B**; c) IONP@SiO_2_-acac/(Tb/Eu)_9_ **2A**; d) IONP@SiO_2_-acac/(Tb/Eu)_9_ **2B**; c) IONP@SiO_2_-acac/(Tb/Eu)_9_@SiO_2_ **3A**; c) IONP@SiO_2_-acac/(Tb/Eu)_9_@SiO_2_ **3B**. The red line represents the Gaussian fit.


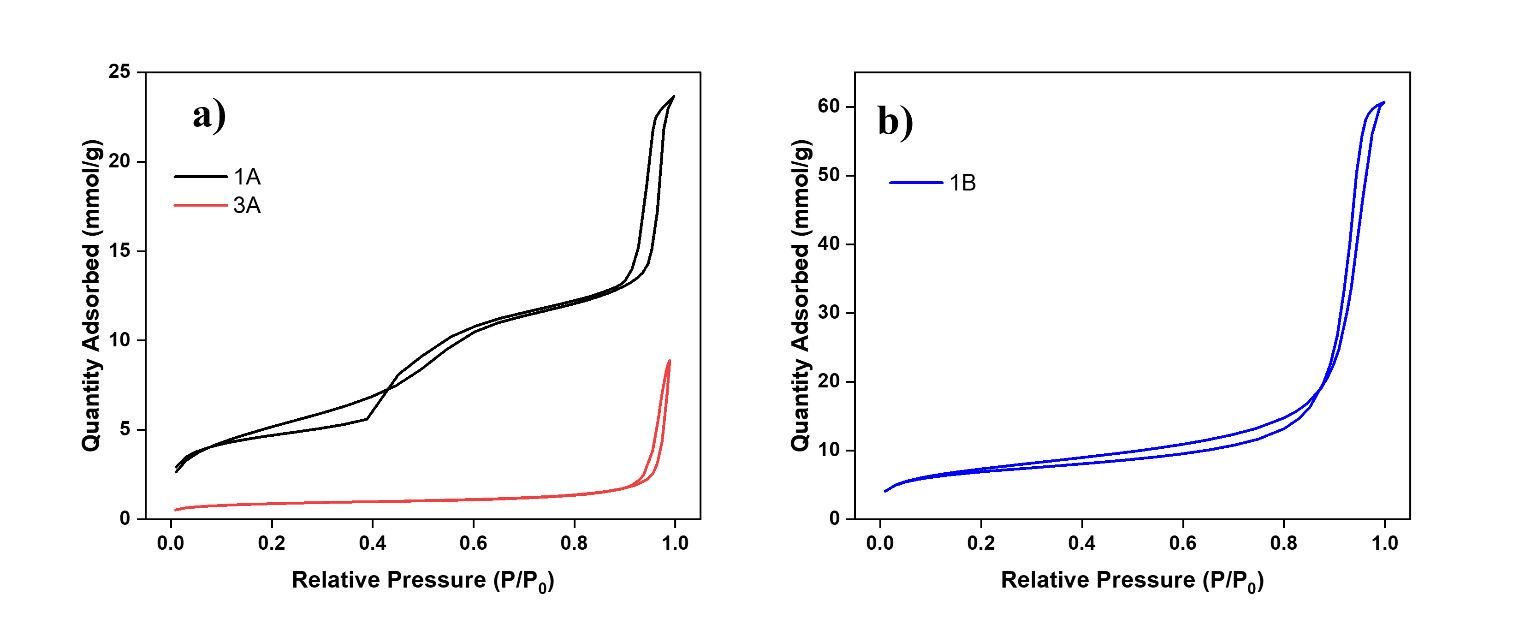


## **Figure S4.** Nitrogen adsorption isotherms for samples: a)**1A** (black) and **3A** (red); b) **1B** (blue).


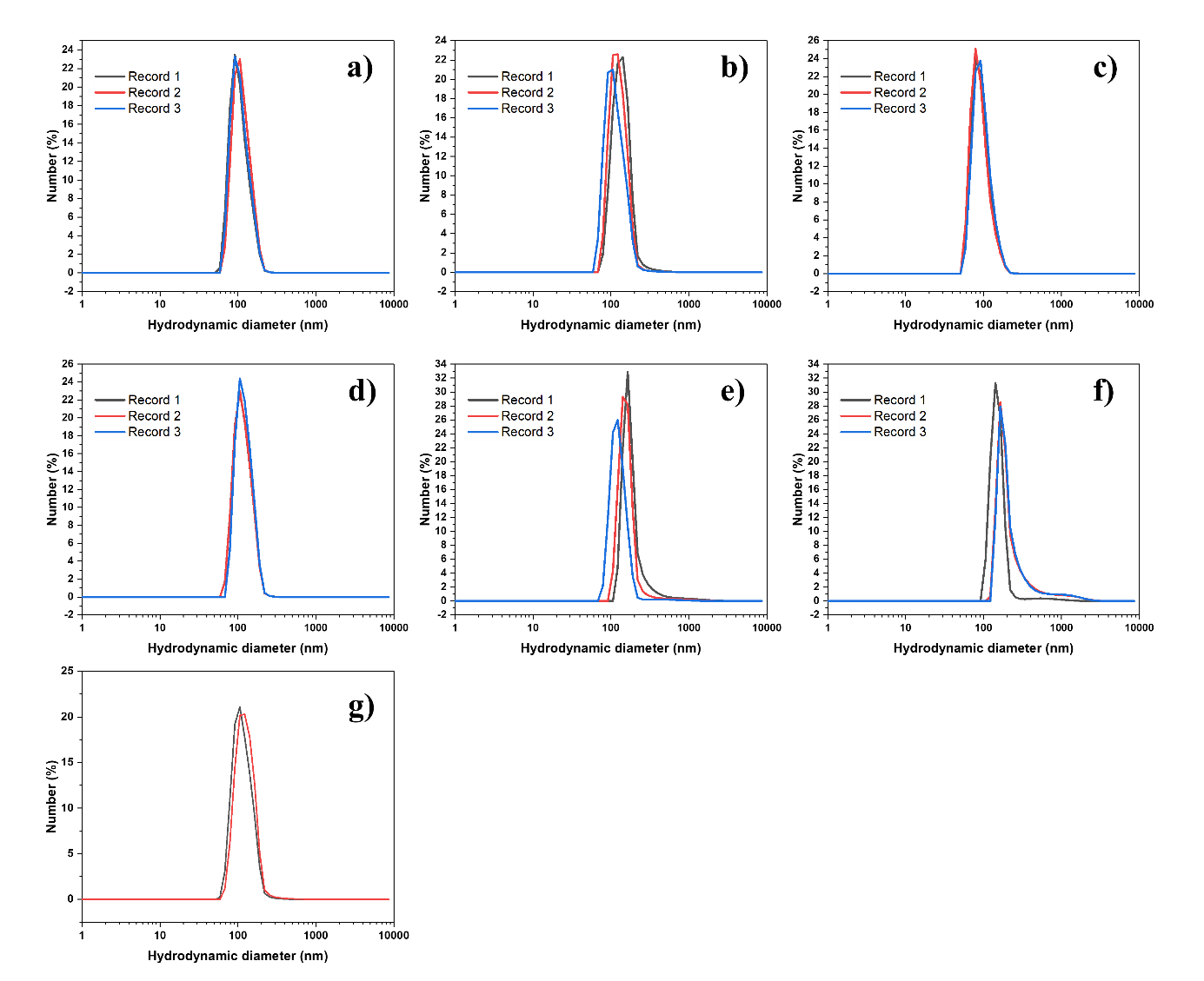


## Figure S5. Hydrodynamic diameter distribution for **1A** (a), **2A** (b), **3A** (c), **1B** (d), **2B** (e) and **3B** (f); g) Comparison of hydrodynamic distributions of sample **3A** freshly prepared and after 27 days of storage in water.


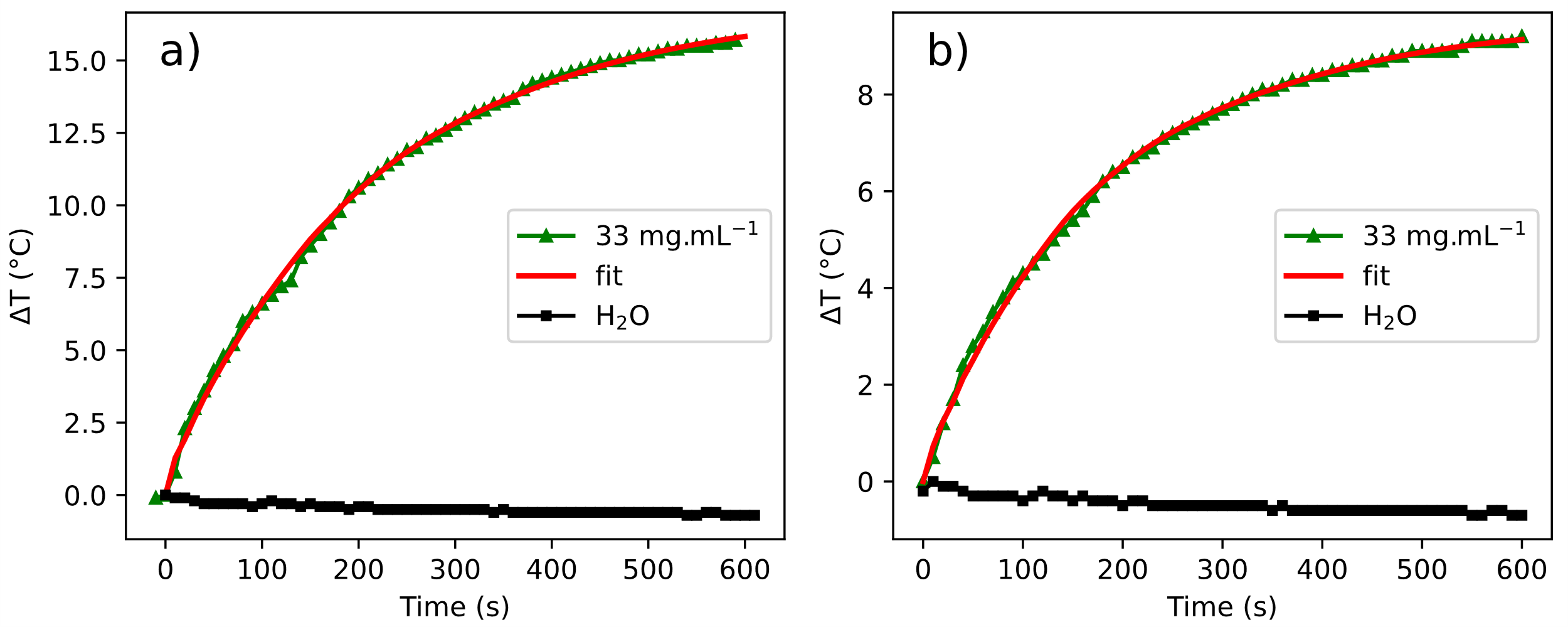


## Figure S6. a) T vs time curves performed for aqueous colloidal solutions of **3A** (a) and **3B** (b) with 9.1 mg·mL^−1^ of IONP performed under irradiation at 808 nm (2.5 W·cm^−2^). The red curves represent the theoretical fit that was employed to extract the photothermal conversion efficiency. The solving process is delineated in section 2.4 of the ESI.

**
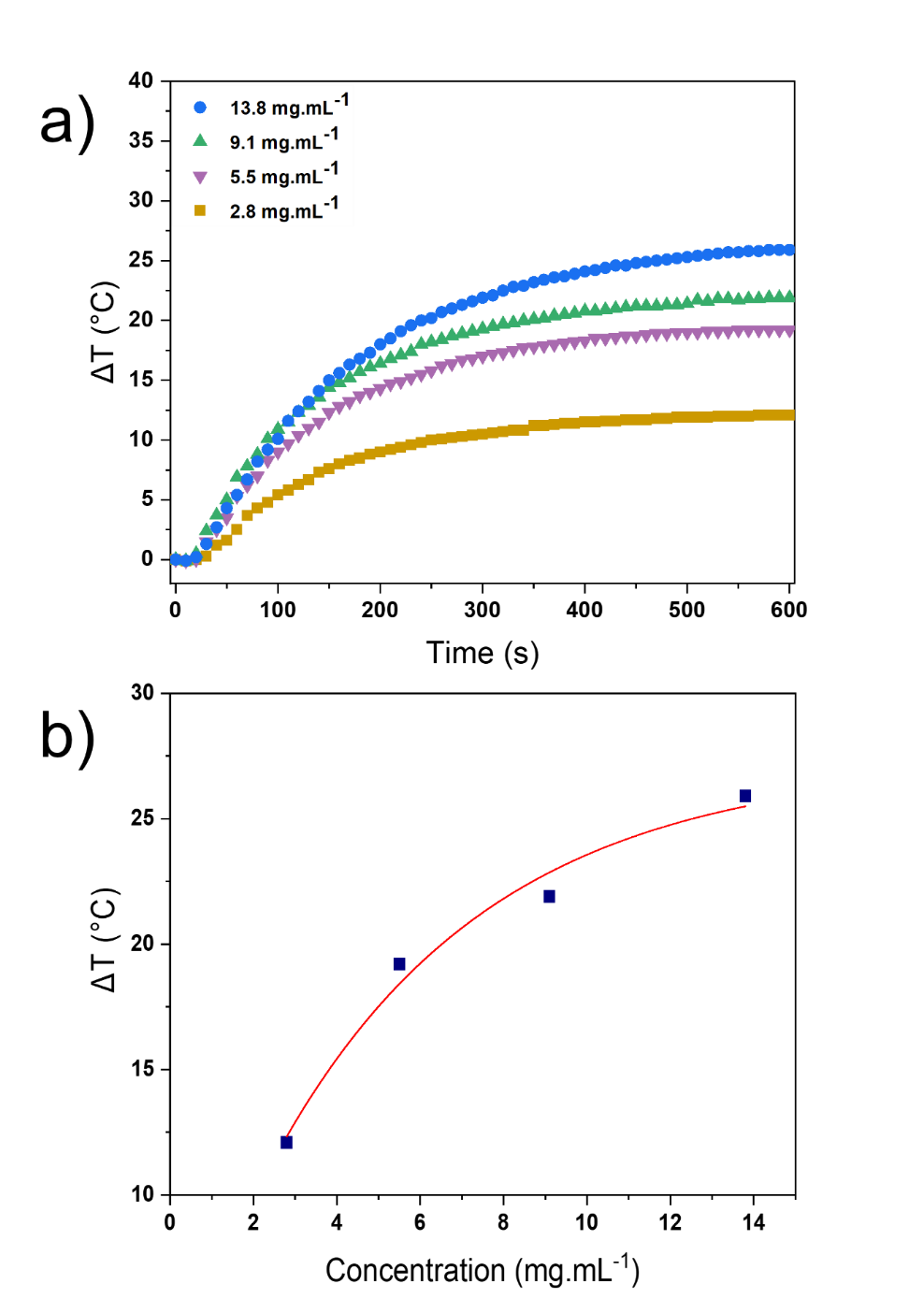
**

## Figure S7. a) ΔT vs time curves performed for aqueous colloidal solutions of IONPs coated with gallic acid with different concentrations of Fe_3_O_4_ performed under irradiation at 808 nm (2.5 W cm^-2^) with ΔT representing the difference between the temperatures of colloidal solutions and water as a reference; b) Corresponding variation of ΔT vs IONPs concentration.

**
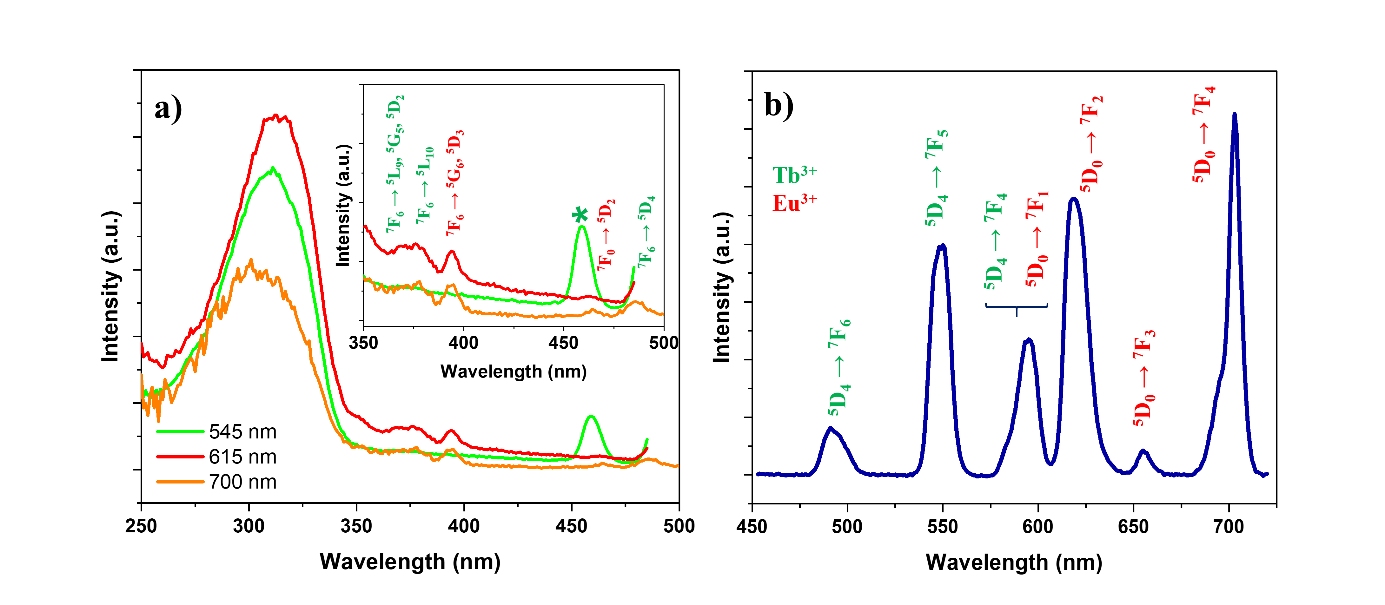
**

## Figure S8. a) Room temperature excitation spectra of IONP@SiO_2_-acac/(Tb/Eu)_9_@SiO_2_ **3B** monitored at λ_em_ = 545 nm (green), 615 nm (red) and 700 nm (orange) in water; b) Room temperature emission spectra of IONP@SiO_2_-acac/(Tb/Eu)_9_@SiO_2_ **3B** under excitation at 315 nm recorded from 450 to 720 nm in water. * Raman scattering peak of water.


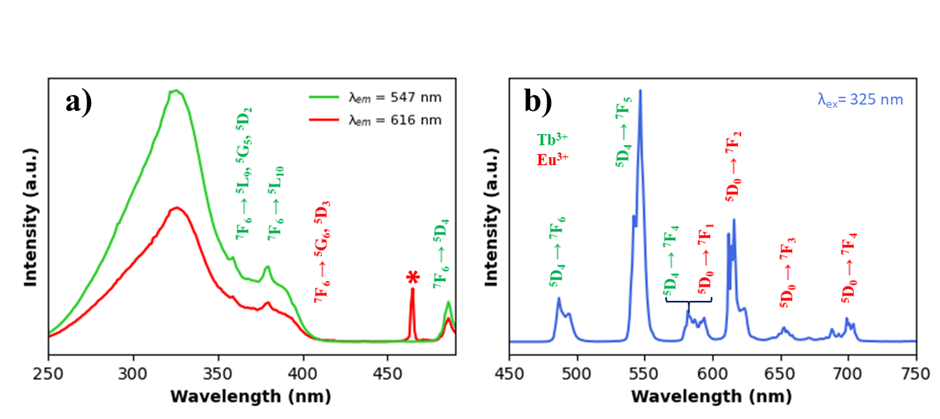


## Figure S9. a) Excitation spectra of [(Tb/Eu)_9_(acac)_16_(μ_3_-OH)_8_(μ_4_-O)(μ_4_-OH)]·H_2_O complex monitored at λ_em_ = 545 (green) and 615 nm (red) measured at room temperature in solid state, b) Emission spectra of [(Tb/Eu)_9_(acac)_16_(μ_3_-OH)_8_(μ_4_-O)(μ_4_-OH)] H_2_O complex performed with λ_ex_ = 328 nm at room temperature in solid state.

**
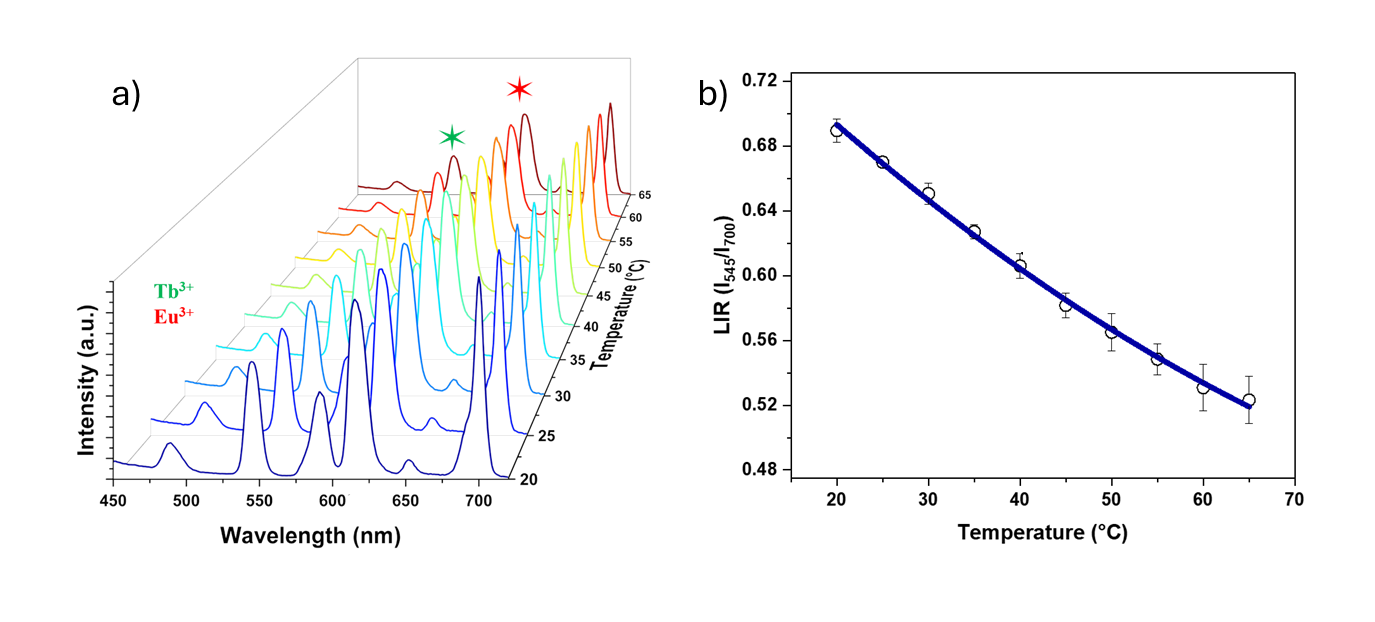
**

## Figure S10. a) Emission spectra of **3B** (λ_ex_ = 315 nm) recorded in water at temperatures from 20 to 65 °C, showing the progressive decrease of Tb^3+^ intensities. b) Luminescence intensity ratio (LIR = I_545_/I_700_) between the Tb^3+^ ^5^D_4_→^7^F_5_ and Eu^3+^ ^5^D_0_→^7^F_4_ transitions plotted against temperature for **3B**. The solid line is a guide to the eye; error bars denote the standard error of the mean from three consecutive temperature cycles.


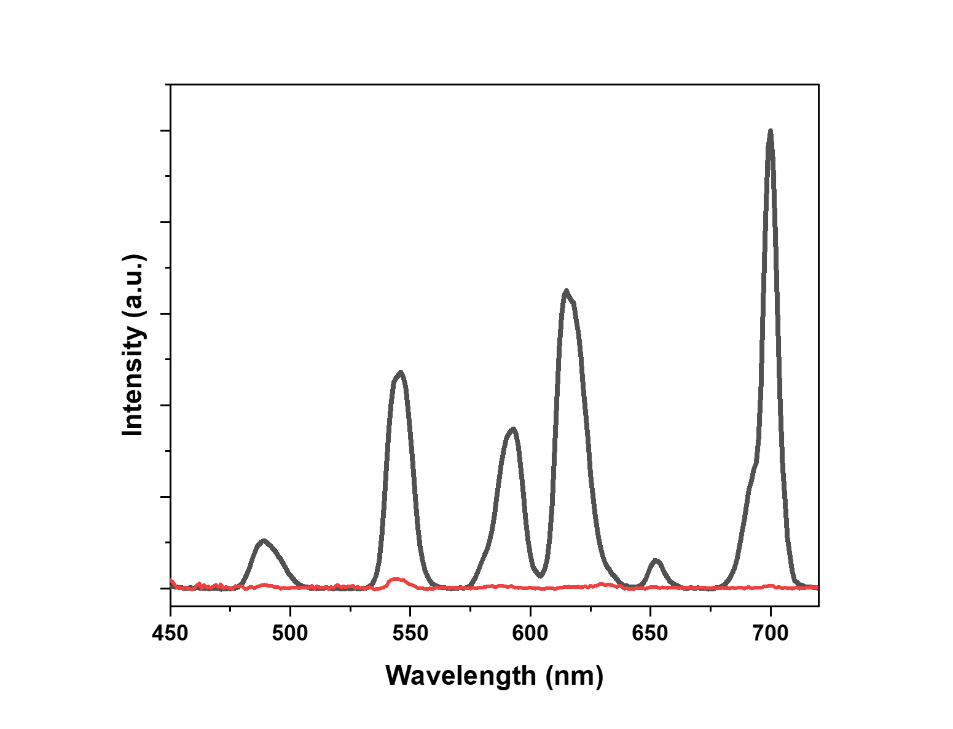


## Figure S11. Room temperature emission spectra under excitation at 315 nm in water for IONP@SiO_2_-acac/(Tb/Eu)_9_@SiO_2_ **3A** (black) and for the supernatant solution of **3A** remaining after heating at 60 °C (red) and removal of the nanoparticles performed in the same conditions.


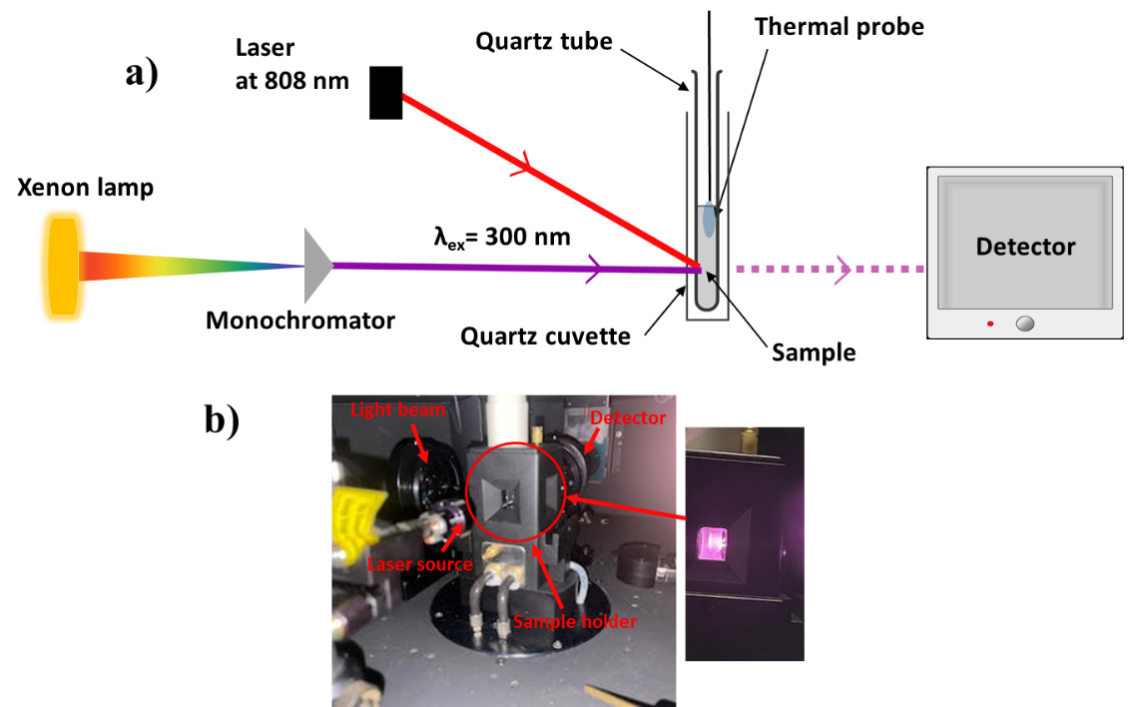


## **Figure S12.** Schematic representation (a) and a photograph (b) of the setup for the luminescence monitoring during the photothermal heating.


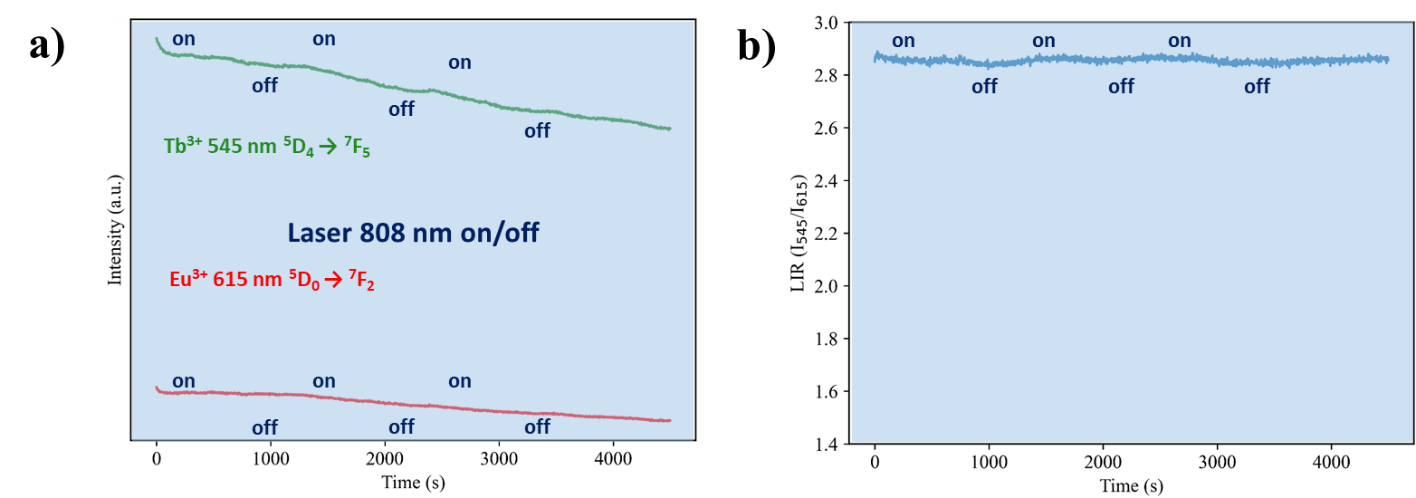


## Figure S13. a) Emission peaks at 545 and 615 nm of SiO_2_-acac/(Tb/Eu)_9_@SiO_2_ nanoparticles (λ_ex_ = 300 nm) recorded as a function of time with temperature fixed at 37 °C; b) LIR (between the ^5^D_4_→^7^F_5_ (Tb^3+^) and ^5^D_0_→^7^F_2_ (Eu^3+^) transitions vs time for SiO_2_-acac/(Tb/Eu)_9_@SiO_2_ nanoparticles.


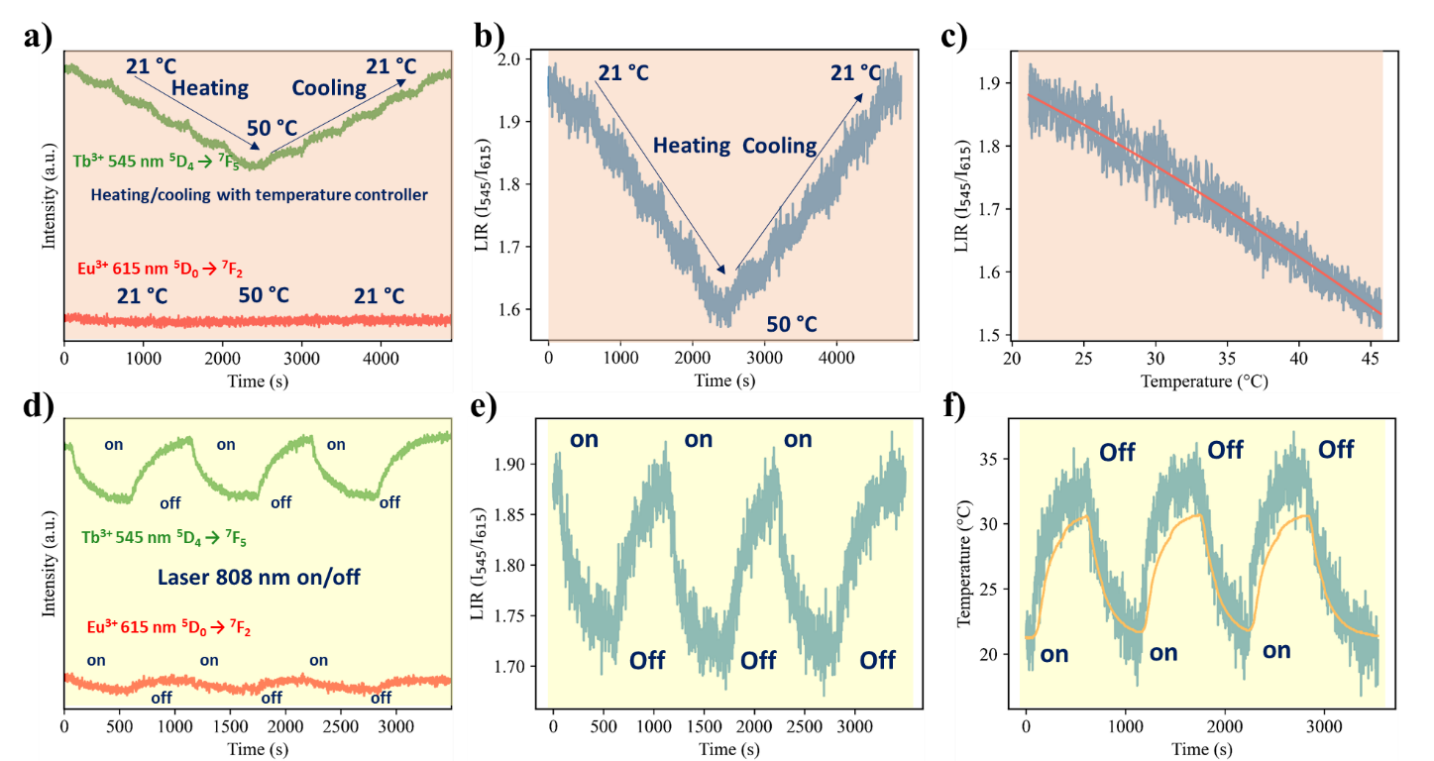


## Figure S14**.** a) Temperature dependences of the emission intensities for ^5^D_4_→^7^F_5_ (Tb^3+^) (green) and ^5^D_0_→^7^F_2_ (Eu^3+^) (orange) transitions during the heating up to 50 °C and cooling of the sample **3A** to 20 °C regulated by the temperature controller with the thermalisation at 20 °C (the same scale is used for Tb^3+^ and Eu^3+^ related intensities); b) Temperature dependence of LIR (between intensities of ^5^D_4_→^7^F_5_ (Tb^3+^) and ^5^D_0_→^7^F_2_ (Eu^3+^) transitions) during the heating up to 50 °C and cooling of the sample to 20 °C regulated by the temperature controller at 20 °C; c) The corresponding calibration curve represented as LIR (I_545_/I_615_) vs temperature (blue) and the macroscopic temperature monitored by optical fibre (orange); d) The fluctuation of the emission intensities of ^5^D_4_→^7^F_5_ (Tb^3+^) (green) and ^5^D_0_→^7^F_2_ (Eu^3+^) (red) transitions under light irradiation at 808 nm (power 2.5 W·cm^−2^) in the on/off mode; e) Fluctuation of LIR (between intensities of ^5^D_4_→^7^F_5_ (Tb^3+^) and ^5^D_0_→^7^F_2_ (Eu^3+^) transitions) under light irradiation at 808 nm in the on/off mode (power 2.5 W·cm^−2^); f) Temperature variation obtained by using the calibration curve under light irradiation at 808 nm in the on/off mode (power 2.58 W·cm^−2^) (blue) and the macroscopic temperature monitored by optical fibre (orange).

# THEORETICAL MODELLING

## 1. Multiphonon, radiative rates, and lifetimes

The multiphonon rates $W_{mp}$ involving the emitting levels (Eu^3+^ ^5^D_0_→^7^F_6_ and Tb^3+^ ^5^D_4_→^7^F_0_) can be estimated from the energy gap law as, ^[10–13]^

| $W_{mp}=W_{0}e^{-\left( \alpha\cdot\Delta E \right)}$ | (S1) |
| --- | --- |

where $\Delta E\cong$ 14,700 and 12,400 cm^−1^ are the ^5^D_4_→^7^F_0_ and ^5^D_0_→^7^F_6_ energy differences and $W_{0}={10}^{8}$ s^−1^ is the decay rate extrapolated to zero energy gap ($\Delta E\approx0$).^[14]^ The quantity $\alpha$ (in cm) depends on the material, and particularly on the phonon energies associated with promoting modes.

According to Miyakawa-Dexter approach,^[15]^ this factor can be estimated by

| $\alpha=\frac{1}{\hbar\bar{\omega}}\left[ \ln\left( \frac{N}{S\left[ n\left( T \right)+1 \right]} \right)-1 \right]$ | (S2) |
| --- | --- |

where $N$ (${=\Delta E}/{\hbar\bar{\omega}}$) is the number of thermally generated phonons, $S$ is the Huang-Rhys factor whose typical values are low for trivalent lanthanides ions (in the order of 0.01 to 0.1) ^[16–18]^. The $n\left( T \right)$ is the thermally averaged phonon occupancy number, given by a Bose-Einstein distribution:

| $n\left( T \right)=\frac{1}{e^{\frac{\hbar\bar{\omega}}{k_{B}T}}-1}$ | (S3) |
| --- | --- |

The vibrational mode chosen as the promoting phonon plays a crucial role in determining the multiphonon decay rate in lanthanide-based systems. In the molecular structure of the {Ln_9_} cluster, ten hydroxyl (O–H) groups are clearly located in close proximity to the Ln^3+^ ions, forming part of the first coordination sphere (Figure S15). These O–H oscillators exhibit high-energy stretching vibrations, typically in the range of 3500–3650 cm^−1^.^[19]^ Thus, O–H stretching modes can be highly efficient in bridging the energy gap between 4f levels of the lanthanide ion via the multiphonon relaxation mechanism. When an excited Ln^3+^ ion undergoes nonradiative decay, the required energy gap $\Delta E$ can be dissipated through the sequential emission of multiple phonons. The number of phonons $N$ required scales inversely with the phonon energy (${N=\Delta E}/{\hbar\bar{\omega}}$), making higher-energy modes such as O–H stretches particularly effective in luminescence quenching process. This is especially relevant for the cases of Tb^3+^ and Eu^3+^ ions, where around four O–H quanta may be needed to bridge their $\Delta E$, compared to more than 10 lower-energy phonons from C–H, C–C, or lattice modes.


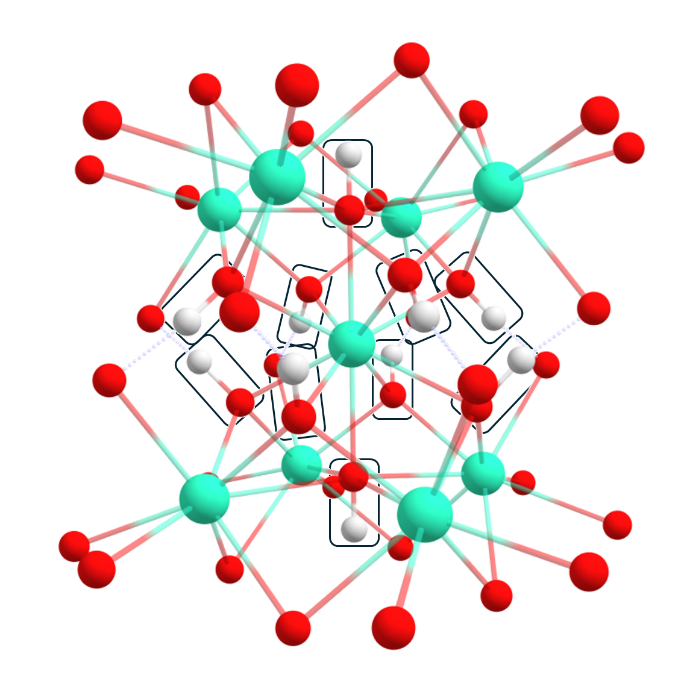


## Figure S15**.** Molecular structure of the {Ln_9_} cluster, highlighting the ten O–H groups that coordinate the nine lanthanide ions. Oxygen atoms are shown in red, lanthanide centers in greenish-blue, and hydrogen atoms of the coordinating hydroxyls in white, illustrating how the ten hydroxide ligands may interact with Ln^3+^.

From the set of Judd-Ofelt intensity parameters estimated in Reference ^[20]^, we can estimate the radiative decay ($A_{rad}$) as the sum of the ^5^D_J‘_→^7^F_J_ (with J‘ = 0 and 4 for Eu^3+^ and Tb^3+^, respectively) individual contributions (J = 0–6):

| $A_{rad}=\sum_{J} A_{J^{'}\to J}=\sum_{J} \frac{{4\left( \omega_{J^{'}\to J} \right)}^{3}}{3\hbar c^{3}\left( 2J^{'}+1 \right)}\left[ \chi\overset{S_{ED}}{\overbrace{e^{2}\sum_{\lambda} \Omega_{\lambda}\left\vert\left\langle4f,{}^{5}{D_{J^{'}}} \right.\left\Vert U^{\left( \lambda\right)} \right\Vert\left. 4f,{}^{7}{F_{J}} \right\rangle\right\vert^{2}}}+n^{3}\underset{S_{MD}}{\underbrace{\left( \mu_{B} \right)^{2}\left\vert\left\langle4f,{}^{5}{D_{J^{'}}} \right.\left\Vert L+g_{s}S \right\Vert\left. 4f,{}^{7}{F_{J}} \right\rangle\right\vert^{2}}} \right]$ | (S4) |
| --- | --- |

where $\omega_{J^{'}\to J}$ are the angular frequency of the transition, $c$ is the speed of light, $e$ is the eletron charge, $\mu_{B}$ is the Bohr magnetion, and $\chi=n\left( n^{2}+2 \right)^{2}/9$ is the Lorentz local field correction, where $n$ is the index of refraction. $S_{ED}$ and $S_{MD}$ are the electric and magnetic dipole strengths and their respective matrix elements can be found in references ^[21,22]^.

Table S1 lists the spectroscopic parameters and phonon‐related constants used to calculate the radiative and multiphonon rates that determine the lifetime $\tau$. Although Eu^3+^ has a larger radiative rate (263 s^−1^) than Tb^3+^ (178 s^−1^), as evienced by its higher intensity in Figure 4b, the calculated multiphonon deactivation (626 s^−1^) is also higher, leading to a shorter overall lifetime (1.12 ms vs. 1.50 ms for Tb^3+^).

## Table S1. Radiative and multiphonon parameters to obtain the lifetime $\tau$.

| Parameter | Eu^3+^ | Tb^3+^ |
| --- | --- | --- |
| $\Omega_{2}$ (×10^−20^ cm^2^) | 4.0 | 3.5 |
| $\Omega_{4}$ (×10^−20^ cm^2^) | 5.9 | 4.4 |
| $\Omega_{6}$ (×10^−20^ cm^2^) | 1.9 | 1.2 |
| $A_{rad}$ (s^−1^) | 263 | 178 |
| $\alpha$ (×10^−4^ cm) | 9.66 | 8.32 |
| $W_{mp}$ (s^−1^) | 626 | 488 |
| $\tau$ (×10^−3^ s) | 1.12 | 1.50 |

## 2. Pairwise Tb-to-Eu energy transfer rates

In this section, a theoretical procedure is presented to estimate the Tb-to-Eu energy transfer rates based on the structures obtained in reference ^[20]^ and the Kushida-Malta theory of nonradiative energy transfer between lanthanide ions.^[23,24]^

The pairwise energy transfer rates between lanthanide ions were calculated, considering the dipole-dipole ($W_{d-d}$), dipole–quadrupole ($W_{d-q}$), quadrupole–quadrupole ($W_{q-q}$), and exchange ($W_{ex}$) mechanisms,^[22,25]^ according to Eqs. S5–S8, respectively:^[26]^

| $W_{d-d}=\frac{\pi}{3\hbar}\frac{S_{d}^{D}S_{d}^{A}}{R^{6}}F$ | (S5) |
| --- | --- |
| $W_{d-q, q-d}=\frac{\pi}{\hbar}\frac{\left( S_{d}^{D}S_{q}^{A}+S_{q}^{D}S_{d}^{A} \right)}{R^{8}}F$ | (S6) |
| $W_{q-q}=\frac{28\pi}{5\hbar}\frac{S_{q}^{D}S_{q}^{A}}{R^{10}}F$ | (S7) |
| $W_{ex}=\frac{2\pi}{\hbar}\left[ \left( \frac{e^{2}}{R} \right)\rho_{f-f}^{2} \right]^{2}F$ | (S8) |

where the quantities $S_{d}$ (in units of esu^2^∙cm^2^) and $S_{q}$ (in units of esu^2^∙cm^4^) are the transition electric dipole and quadrupole strengths, respectively:

| $S_{d}^{x}=\frac{2e^{2}\left( 1-\sigma_{1}^{x} \right)^{2}}{\left( 2J_{x}+1 \right)}\sum_{\lambda} \Omega_{\lambda}^{x}{\left\langle\psi_{x}J_{x} \right.\left\Vert U^{\left( \lambda\right)} \right\Vert\left. \psi_{x}^{*}J_{x}^{*} \right\rangle}^{2}$ | (S9) |
| --- | --- |
| $S_{q}^{x}=\frac{e^{2}\left( 1-\sigma_{2}^{x} \right)^{2}}{\left( 2J_{x}+1 \right)}{\left\langle f \right.\left\Vert C^{\left( 2 \right)} \right\Vert\left. f \right\rangle}^{2}\left\langle r^{2} \right\rangle_{x}^{2}{\left\langle\psi_{x}^{*}J_{x}^{*} \right.\left\Vert U^{\left( 2 \right)} \right\Vert\left. \psi_{x}J_{x} \right\rangle}^{2}$ | (S10) |

With index x = A for the acceptor and x = D for the donor, $J_{x}$ is the total angular momentum quantum number of the excited state of the donor or the ground state of the acceptor. $R$ is the donor acceptor distance, i.e., the distance between Tb^3+^ and Eu^3+^ centers. $\left( 1-\sigma_{1}^{x} \right)$ and $\left( 1-\sigma_{2}^{x} \right)$ are the 4f orbitals shielding factors for the electric dipole and quadrupole interactions, which were estimated from density functional theory calculations on the orbitals overlap integrals.^[27]^

In the context of energy transfer, the $\Omega_{\lambda}$ values represent the forced electric dipole (FED) contributions to the Judd–Ofelt intensity parameters. These are smaller than the total $\Omega_{\lambda}$ reported in Table S1 because the dynamic coupling mechanism—which strongly influences Eu^3+^ and Tb^3+^ ions—cannot be included here. Previous calculations yielded the following $\Omega_{\lambda}$ (FED) values (in 10^−20^ cm^2^): for Tb^3+^ $[\Omega_{2}=0.05; \Omega_{4}= 0.06; \Omega_{6}=0.12]$ and for Eu^3+^ $[\Omega_{2}=0.07; \Omega_{4}=0.11; \Omega_{6}=0.24]$.^[20]^ The $W_{q-q}$ and $W_{ex}$ mechanisms are independent of the intensity parameters.

In Eq. S8, $\rho_{f-f}$ represents the overlap integral between 4f subshells of the donor and acceptor lanthanide ions. The values of $\rho_{f-f}$ as a function of the Tb–Eu distance ($R_{L}$) were obtained using the parametric function $\rho_{f-f}\left( R \right)=exp\left( a+bR+cR^{2} \right)$, with $a=-0.032$, $b=-0.261$, and $c=-0.341$ for the Tb-Eu pair.^[27]^ The $\rho_{f-f}$ decreases rapidly to zero with the increase of the donor-acceptor distance $R$, as demonstrated in reference^[25]^ for the case of Tb–Eu. This is why the $W_{ex}$ term can often be neglected in the Ln–Ln energy transfer processes, where the donor-acceptor distances are typically higher than 4 Å.^[24]^

In all equations related to Ln-Ln energy transfer mechanisms, the spectral overlap factor ($F$) is involved. This quantity is associated with the energy mismatch between the donor and acceptor states, and the following expression for $F$ has been used:^[24]^

| $F=\frac{ln(2)}{\sqrt{\pi}}\frac{1}{\hbar^{2}\gamma_{Tb}\gamma_{Eu}}\left\{ \left[ \left( \frac{1}{\hbar\gamma_{Tb}} \right)^{2}+\left( \frac{1}{\hbar\gamma_{Eu}} \right)^{2} \right]ln(2) \right\}^{-\frac{1}{2}}\times exp\left[ \frac{1}{4}\frac{\left( \frac{2 \Delta}{\left( \hbar\gamma_{Tb} \right)^{2}}ln2 \right)^{2}}{\left[ \left( \frac{1}{\hbar\gamma_{Eu}} \right)^{2}+\left( \frac{1}{\hbar\gamma_{Tb}} \right)^{2} \right]ln2}-\left( \frac{\Delta}{\hbar\gamma_{Tb}} \right)^{2}ln(2) \right]$ | (S11) |
| --- | --- |

where $\hbar\gamma_{Tb}$ and $\hbar\gamma_{Eu}$ correspond to the bandwidths at half-height of the Tb^3+^ (donor) and Eu^3+^ (acceptor) transitions, respectively. $\Delta$ is the energy difference between donor and acceptor transitions ($\Delta E=E_{D}-E_{A}$). In the present work, $\hbar\gamma_{Tb}$= $\hbar\gamma_{Eu}$=300 cm^−1^ is considered, a value acceptable concerning the narrow nature of 4f transitions.

The energy transfer pathways were chosen with the combination of 210 donor transitions from Tb^3+^ and 30 acceptor transitions (Eu^3+^: ^7^F_0,1_→ ^5^D_J_/^5^L_J_/^5^G_J_), totalising 6300 pathways. The selection rules on the *J* quantum numbers (|*J – J'*| ≤ λ ≤ *J + J'*) were taken into account for multipolar mechanisms ($W_{d-d}$, $W_{d-q}$, and $W_{q-q}$) while no defined selection rules on *J* appear for the exchange mechanism ($W_{ex}$).^[28]^ The magnetic dipole-magnetic dipole mechanism was not considered due to its low participation in short-range processes in comparison to the $W_{q-q}$ and $W_{ex}$.^[28]^ As an example of the complexity of the calculations, the 6300 forward and 6300 backward energy transfer pathways for the shortest distance (R = 3.439 Å) at 65 °C were provided in the Resume_ET.xlsx (Supplementary File).

For each pathway, the energy transfer rates were calculated by the sum over Eqs. S5–S8, i.e., $\omega=W_{d-d}+W_{d-q}+W_{q-q}+W_{ex}$. Based on the Jablonski–Perrin diagram for each channel (Figure 4d), we can distinguish the individual contributions of the different energy transfer mechanisms as a function of temperature. In other words, the energy transfer is analyzed between specific states (or groups of states) as indicated in kets in Figure 4d.

## 3. Effective Tb-to-Eu energy transfer rates

Once the pairwise energy transfer (ET) rates between Tb^3+^ and Eu³⁺ ions are calculated, a Monte Carlo simulation can be used to model the distribution of these ions in the host matrix. This simulation considers only the interaction between individual Tb–Eu pairs and was implemented using a homemade C-language program. To mimic effective energy transfer in the material, the unit cell (1×1×1) was expanded into a larger supercell comprising 2,000 {Tb_9_} clusters. Eu^3+^ ions were randomly substituted into this matrix until the desired concentration was reached—specifically maintaining an experimental Eu/Tb ratio of 1:9.

From these simulations, the occurrence coefficients $O_{i}\left( x \right)$ for Tb–Eu pairs can be obtained as a function of the acceptor (Eu^3+^) ratio $x$, for specific donor–acceptor distances. In this system, the smallest relevant distances are $R_{i}$ = 3.439, 3.440, 3.517, 3.548, and 4.959 Å (Figure S16),^[20]^ corresponding to the complex incorporated into an iron oxide core–silica shell nanostructure.

Thus, the average forward $\left\langle W \right\rangle_{odd\to even}$ and backward $\left\langle W \right\rangle_{even\to odd}$ energy transfer rates (see Figure 4d) can then be calculated using:^[22]^

| $\left\langle W \right\rangle_{odd\to even}=\left( 1-x \right)x\left( \sum_{i} O_{i}\left( x \right)\omega_{i}^{f} \right)$ | (S12) |
| --- | --- |
| $\left\langle W \right\rangle_{even\to odd}=\left( 1-x \right)x\left( \sum_{i} O_{i}\left( 1-x \right)\omega_{i}^{b} \right)$ | (S13) |

where $\omega_{i}^{f}$ and $\omega_{i}^{b}$ are the pairwise forward and backward energy transfer rates at the 𝑖-th Tb–Eu distance $R_{i}$, respectively. The acceptor and donor stoichiometries are represented by $x$ and $1-x$, and the coefficients $O_{i}\left( x \right)$ and $O_{i}\left( 1-x \right)$ describe the occurrence of forming a Tb–Eu pair at distance $R_{i}$, with respect to the Eu^3+^ and Tb^3+^ ions, respectively. These coefficients are defined as:

| $O_{i}\left( x \right)=\frac{\mathcal{N}\left( i \right)}{s\cdot x} , O_{i}\left( 1-x \right)=\frac{\mathcal{N}\left( i \right)}{s\cdot\left( 1-x \right)}$ | (S14) |
| --- | --- |

where $\mathcal{N}\left( i \right)$ is the number of Tb–Eu pairs at distance $R_{i}$, and $s$ is the total number of host sites (2,000 clusters = 18,000 Ln^3+^ sites). Since the backward energy transfer proceeds from Eu^3+^ to Tb^3+^, Tb^3+^ acts as the acceptor in this case, justifying the use of $O_{i}\left( 1-x \right)$ in the expression for $\left\langle W \right\rangle^{b}$.^[22]^ These coefficients represent how many acceptors a donor can access at a given distance.

The final values of $O_{i}\left( x \right)$ and $O_{i}\left( 1-x \right)$ were obtained from simulations and are presented in Table S2, while the values of $\left\langle W \right\rangle_{odd\to even}$ and $\left\langle W \right\rangle_{even\to odd}$ as a function of temperature are shown in Figures 4e-4h.


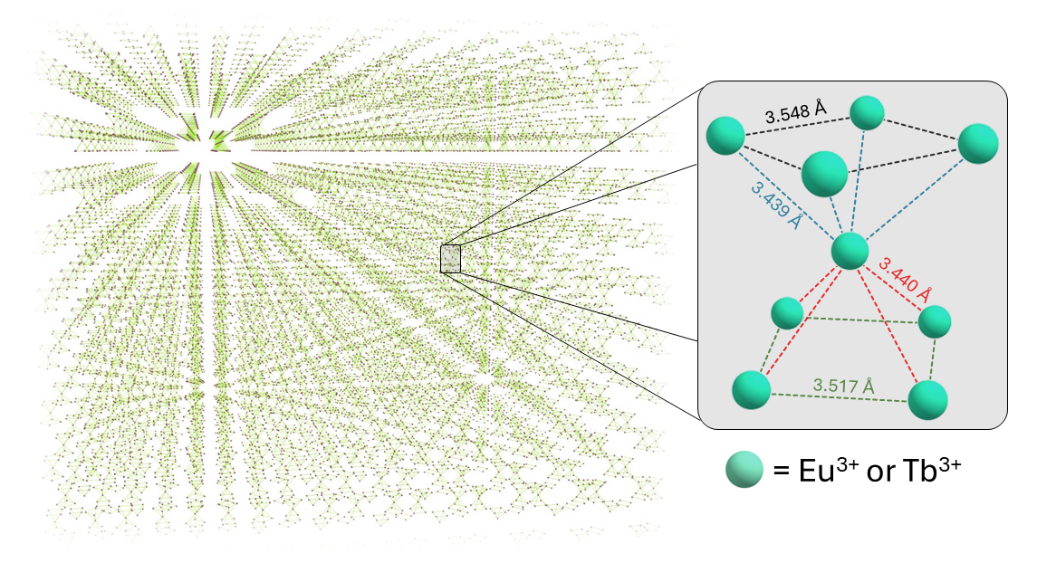


## Figure S16**.** Expanded supercell representation showing only the {Ln_9_} clusters. The distances between each lanthanide ion within a single {Ln_9_} cluster (e.g., 3.439 Å, 3.440 Å, 3.517 Å, and 3.548 Å) are illustrated.

## Table S2. Simulated coefficients of occurrences for a Eu^3+^/Tb^3+^ ratio of 1:9 for the five shortest Tb^3+^-Eu^3+^ distances.

| $R$ (Å) | $O_{i}\left( x \right)$ | $O_{i}\left( 1-x \right)$ |
| --- | --- | --- |
| 3.439 | 0.790 | 0.099 |
| 3.440 | 0.789 | 0.098 |
| 3.517 | 0.714 | 0.089 |
| 3.548 | 0.793 | 0.099 |
| 4.959 | 0.396 | 0.049 |

## 4. Rate equations modelling and thermometric simulation

In the previous sections, we have calculated all the necessary rates (energy transfer and decay rates) to insert into a rate equation model. By solving this system ordinary differential equations (ODEs) numerically, we can estimate the steady-state populations of the emitting levels Eu^3+^ ^5^D_0_ and Tb^3+^ ^5^D_4_. Considering the level scheme depicted in Figure 4d, we construct the following set of ODEs:

| ODE | Level |  |
| --- | --- | --- |
| $\frac{d}{dt}P_{0}^{Eu}\left( t \right)=\frac{1}{\tau_{Eu}}P_{2}\left( t \right)-\phi\cdot P_{0}^{Eu}\left( t \right)+\gamma\left( P_{0}^{Tb}\left( t \right)-P_{0}^{Eu}\left( t \right) \right)$ | Ground state of Eu^3+^ | (S15) |
| $\frac{d}{dt}P_{0}^{Tb}\left( t \right)=\frac{1}{\tau_{Tb}}P_{1}\left( t \right)-\phi\cdot P_{0}^{Tb}\left( t \right)+\gamma\left( P_{0}^{Eu}\left( t \right)-P_{0}^{Tb}\left( t \right) \right)$ | Ground state of Tb^3+^ | (S16) |
| $\frac{d}{dt}P_{1}\left( t \right)=W_{3\to1}\cdot P_{3}\left( t \right)+\left\langle W \right\rangle_{2\to1}\cdot P_{2}\left( t \right)+\left\langle W \right\rangle_{4\to1}\cdot P_{4}\left( t \right)-\left( \left\langle W \right\rangle_{1\to2}+\left\langle W \right\rangle_{1\to4}+\frac{1}{\tau_{Tb}} \right)P_{1}\left( t \right)$ | Tb^3+^ ^5^D_4_ | (S17) |
| $\frac{d}{dt}P_{2}\left( t \right)=W_{4\to2}\cdot P_{4}\left( t \right)+\left\langle W \right\rangle_{1\to2}\cdot P_{1}\left( t \right)+\left\langle W \right\rangle_{3\to2}\cdot P_{3}\left( t \right)-\left( \left\langle W \right\rangle_{2\to3}+\left\langle W \right\rangle_{2\to1}+\frac{1}{\tau_{Eu}} \right)P_{2}\left( t \right)$ | Eu^3+^ ^5^D_0_ | (S18) |
| $\frac{d}{dt}P_{3}\left( t \right)=\phi\cdot P_{0}^{Tb}\left( t \right)+\left\langle W \right\rangle_{4\to3}\cdot P_{4}\left( t \right)+\left\langle W \right\rangle_{2\to3}\cdot P_{2}\left( t \right)-\left( W_{3\to1}+\left\langle W \right\rangle_{3\to4}+\left\langle W \right\rangle_{3\to2} \right)P_{3}\left( t \right)$ | Tb^3+^  ^5^D_3_–^5^F_5_ | (S19) |
| $\frac{d}{dt}P_{4}\left( t \right)=\phi\cdot P_{0}^{Eu}\left( t \right)+\left\langle W \right\rangle_{3\to4}\cdot P_{3}\left( t \right)+\left\langle W \right\rangle_{1\to4}\cdot P_{1}\left( t \right)-\left( W_{4\to2}+\left\langle W \right\rangle_{4\to3}+\left\langle W \right\rangle_{4\to1} \right)P_{4}\left( t \right)$ | Eu^3+^  ^5^D_1_–^5^L_10_ | (S20) |

In these expressions, $P_{0}^{Eu}\left( t \right)$ and $P_{0}^{Tb}\left( t \right)$ denote the time-dependent populations of the Eu^3+^ and Tb^3+^ ground states, respectively. The variables $P_{1}\left( t \right)$ and $P_{2}\left( t \right)$ represent the populations of the Tb^3+^ ^5^D_4_ and Eu^3+^ ^5^D_0_ emitting levels. The combined manifolds Tb^3+^ ^5^D_3_–^5^F_5_ and Eu^3+^ ^5^D_1_–^5^L_10_ are represented by $P_{3}\left( t \right)$ and $P_{4}\left( t \right)$, respectively. The parameters $\tau_{Tb}$ and $\tau_{Eu}$ are the intrinsic lifetimes of the Tb^3+^ ^5^D_4_ and Eu^3+^ ^5^D_0_ levels (accounting for both radiative and multiphonon decay), as represented in Table S1. The $\phi=100$ s denotes the effective photo-excitation rate into the higher excited manifolds (typically in the same order of the $A_{rad}$), while $\left\langle W \right\rangle_{i\to j}$ refers to the averaged energy transfer rate from level $i$ to level $j$. In addition, $W_{3\to1}$ and $W_{4\to2}$ (both set to 10^6^ s^−1^)^[29]^ are the average multiphonon rate ^5^D_3_→^5^D_4_ in Tb^3+^ and ^5^D_1_→^5^D_0_ in Eu^3+^, respectively. Finally, the parameter $\gamma={10}^{14}$ s^−1^ imposes equilibrium between the two ground‐state populations $P_{0}^{Eu}\left( t \right)$ and $P_{0}^{Tb}\left( t \right)$. This large rate prevents any unphysical population inversion (i.e., excited levels exceeding ground‐state populations) while leaving unchanged the ratio $P_{1}/P_{2}$, which defines the theoretical luminescence intensity ratio (LIR).

Solving Eqs. S15–S20 with the Radau method^[30]^ and extracting the steady‐state populations ($dP_{i}\left( t \right)/dt =0$) yields numerical values for $P_{1}$ and $P_{2}$. Their quotient, $P_{1}/P_{2}$, as a function of temperature (via temperature-dependent energy transfer rates), provides the LIR versus temperature. Accordingly, Eqs. S15–S20 are solved at each temperature from 20 °C to 65 °C in 5 °C increments.

## 5. Thermometric performance

The relative thermal sensitivity $S_{r}$ was calculated as

| $S_{r}\left( T \right)=\frac{1}{LIR\left( T \right)}\left\vert\frac{dLIR\left( T \right)}{dT} \right\vert$ | (S21) |
| --- | --- |

where $LIR$ is the thermometric parameter. The derivative $dLIR/dT$ was obtained from a linear fit of the LIR(T) data ($LIR=aT+b$), and the standard error of the fitted slope ($a$) was used as $\sigma_{a}$. The uncertainty in LIR, denoted $\sigma_{LIR}$, corresponds to the standard deviation of three independent measurements at each temperature point. The combined uncertainty in $S_{r}$ was estimated by standard error propagation as:

| $\sigma\left( S_{r} \right)=S_{r}\sqrt{\left( \frac{\sigma_{LIR}}{LIR} \right)^{2}}$ | (S22) |
| --- | --- |

Values of $S_{r}$ and $\sigma\left( S_{r} \right)$ were reported as percentages.

The thermal uncertainty was also calculated as the smallest temperature change that can be measured, *i.e*.:

| $\delta T=\frac{\delta LIR\left( T \right)}{\mathrm{LIR}\left( T \right)\cdot S_{r}\left( T \right)}$ | (S23) |
| --- | --- |

# REFERENCES

[1] B. Yuan, X. He, Y. Chen, K. Wang, “Preparation of Nanosilica/Polynorbornene Nanocomposite by Covalently Immobilized Silica-Supported Acetylacetonate Palladium(II) Dichloride Catalyst” *Macromolecular Chemistry and Physics* **2011**, *212*, 2378–2388.

[2] T. Pelluau, S. Sene, B. Garcia-Cirera, B. Albela, L. Bonneviot, J. Larionova, Y. Guari, “Multifunctionalized Mesostructured Silica Nanoparticles Containing Mn2 Complex for Improved Catalase-Mimicking Activity in Water” *Nanomaterials* **2022**, *12*, 1136.

[3] S. Petit, F. Baril-Robert, G. Pilet, C. Reber, D. Luneau, “Luminescence spectroscopy of europium(III) and terbium(III) penta-, octa- and nonanuclear clusters with β-diketonate ligands” *Dalton Trans.* **2009**, 6809–6815.

[4] W. W. Yu, J. C. Falkner, C. T. Yavuz, V. L. Colvin, “Synthesis of monodisperse iron oxide nanocrystals by thermal decomposition of iron carboxylate salts” *Chem. Commun.* **2004**, 2306–2307.

[5] B. Bouvet, S. Sene, G. Félix, J. Havot, G. Audran, S. R. A. Marque, J. Larionova, Y. Guari, “Cascade strategy for triggered radical release by magnetic nanoparticles grafted with thermosensitive alkoxyamine” *Nanoscale* **2022**, *15*, 144–153.

[6] A. Adam, S. Harlepp, F. Ghilini, G. Cotin, B. Freis, J. Goetz, S. Bégin, M. Tasso, D. Mertz, “Core-shell iron oxide@stellate mesoporous silica for combined near-infrared photothermia and drug delivery: Influence of pH and surface chemistry” *Colloids and Surfaces A: Physicochemical and Engineering Aspects* **2022**, *640*, 128407.

[7] N. Lang, A. Tuel, “A Fast and Efficient Ion-Exchange Procedure To Remove Surfactant Molecules from MCM-41 Materials” *Chem. Mater.* **2004**, *16*, 1961–1966.

[8] A. Hernández Montoto, R. Montes, A. Samadi, M. Gorbe, J. M. Terrés, R. Cao-Milán, E. Aznar, J. Ibañez, R. Masot, M. D. Marcos, M. Orzáez, F. Sancenón, L. B. Oddershede, R. Martínez-Máñez, “Gold Nanostars Coated with Mesoporous Silica Are Effective and Nontoxic Photothermal Agents Capable of Gate Keeping and Laser-Induced Drug Release” *ACS Appl. Mater. Interfaces* **2018**, *10*, 27644–27656.

[9] *COMSOL Multiphysics® v. 6.0. www.comsol.com. COMSOL AB* **2022**.

[10] R. Reisfeld, C. K. Jørgensen, *Lasers and Excited States of Rare Earths*, Springer-Verlag Berlin Heidelberg, **1977**.

[11] H. W. Moos, “Spectroscopic relaxation processes of rare earth ions in crystals” *Journal of Luminescence* **1970**, *1–2*, 106–121.

[12] B. Z. Malkin in *Spectroscopic Properties of Rare Earths in Optical Materials* (Eds.: R. Hull, J. Parisi, R.M. Osgood, H. Warlimont, G. Liu, B. Jacquier), Springer Berlin Heidelberg, Berlin, Heidelberg, **2005**, pp. 130–190.

[13] L. A. Riseberg, M. J. Weber in *Progress in Optics*, **1977**, pp. 89–159.

[14] A. N. Carneiro Neto, E. Kasprzycka, A. S. Souza, P. Gawryszewska, M. Suta, L. D. Carlos, O. L. Malta, “On the long decay time of the ^7^F_5_ level of Tb^3+^” *Journal of Luminescence* **2022**, *248*, 118933.

[15] T. Miyakawa, D. L. Dexter, “Phonon Sidebands, Multiphonon Relaxation of Excited States, and Phonon-Assisted Energy Transfer between Ions in Solids” *Physical Review B* **1970**, *1*, 2961–2969.

[16] N. Yamada, S. Shionoya, T. Kushida, “Phonon-Assisted Energy Transfer between Trivalent Rare Earth Ions” *Journal of the Physical Society of Japan* **1972**, *32*, 1577–1586.

[17] W. H. Fonger, C. W. Struck, “Unified model of energy transfer for arbitrary Franck-Condon offset and temperature” *Journal of Luminescence* **1978**, *17*, 241–261.

[18] F. Auzel, G. F. De Sá, W. M. de Azevedo, “An example of concentration sensitive electron-phonon coupling in {(C_4_H_9_)_4_N}_3_ Eu_x_Y_1−x_(NCS)_6_ and a new hypothesis for self-quenching” *Journal of Luminescence* **1980**, *21*, 187–192.

[19] R. M. Silverstein, F. X. Webster, D. J. Kiemle, *Spectrometric Identification of Organic Compounds*, Wiley, **2005**.

[20] T. Pelluau, S. Sene, L. M. A. Ali, G. Félix, F. Manhes, A. N. Carneiro Neto, L. D. Carlos, B. Albela, L. Bonneviot, E. Oliviero, M. Gary-Bobo, Y. Guari, J. Larionova, “Hybrid multifunctionalized mesostructured stellate silica nanoparticles loaded with β-diketonate Tb^3+^/Eu^3+^ complexes as efficient ratiometric emissive thermometers working in water” *Nanoscale* **2023**, *15*, 14409–14422.

[21] W. T. Carnall, H. Crosswhite, H. M. Crosswhite, *Energy level structure and transition probabilities in the spectra of the trivalent lanthanides in LaF₃*, Argonne, IL, United States, **1978**.

[22] V. Trannoy, A. N. Carneiro Neto, C. D. S. Brites, L. D. Carlos, H. Serier‐Brault, “Engineering of Mixed Eu^3+^/Tb^3+^ Metal‐Organic Frameworks Luminescent Thermometers with Tunable Sensitivity” *Advanced Optical Materials* **2021**, *9*, 2001938.

[23] T. Kushida, “Energy Transfer and Cooperative Optical Transitions in Rare-Earth Doped Inorganic Materials. I. Transition Probability Calculation” *Journal of the Physical Society of Japan* **1973**, *34*, 1318–1326.

[24] O. L. Malta, “Mechanisms of non-radiative energy transfer involving lanthanide ions revisited” *Journal of Non-Crystalline Solids* **2008**, *354*, 4770–4776.

[25] A. N. Carneiro Neto, R. T. Moura, A. Shyichuk, V. Paterlini, F. Piccinelli, M. Bettinelli, O. L. Malta, “Theoretical and Experimental Investigation of the Tb 3+ → Eu 3+ Energy Transfer Mechanisms in Cubic A 3 Tb 0.90 Eu 0.10 (PO 4 ) 3 (A = Sr, Ba) Materials” *The Journal of Physical Chemistry C* **2020**, *10*, 10105–10116.

[26] C. D. S. Brites, R. Marin, M. Suta, A. N. Carneiro Neto, E. Ximendes, D. Jaque, L. D. Carlos, “Spotlight on Luminescence Thermometry: Basics, Challenges, and Cutting‐Edge Applications” *Advanced Materials* **2023**, *35*, 2302749.

[27] A. N. Carneiro Neto, R. T. Moura, “Overlap integrals and excitation energies calculations in trivalent lanthanides 4f orbitals in pairs Ln-L (L = Ln, N, O, F, P, S, Cl, Se, Br, and I)” *Chemical Physics Letters* **2020**, *757*, 137884.

[28] A. N. Carneiro Neto, R. T. Moura, A. Shyichuk, V. Paterlini, F. Piccinelli, M. Bettinelli, O. L. Malta, “Theoretical and Experimental Investigation of the Tb^3+^ → Eu^3+^ Energy Transfer Mechanisms in Cubic A_3_Tb_0.90_Eu_0.10_(PO_4_)_3_ (A = Sr, Ba) Materials” *The Journal of Physical Chemistry C* **2020**, *124*, 10105–10116.

[29] A. De, M. A. Hernández-Rodríguez, A. N. Carneiro Neto, V. Dwij, V. Sathe, L. D. Carlos, R. Ranjan, “Resonance/off-resonance excitations: implications on the thermal evolution of Eu^3+^ photoluminescence” *Journal of Materials Chemistry C* **2023**, *11*, 6095–6106.

[30] E. Hairer, G. Wanner in *Encyclopedia of Applied and Computational Mathematics* (Ed.: B. Engquist), Springer Berlin Heidelberg, Berlin, Heidelberg, **2015**, pp. 1213–1216.
