# Supplementary figures and images for: Photothermal Heating and Real‐Time In Situ Luminescent Thermometry with Iron Oxide Core‐Silica Shell Nano‐Objects
Source: Small. 2025 Oct 22;21(49):e08497. doi: 10.1002/smll.202508497 (PMC12696789; doi:10.1002/smll.202508497)

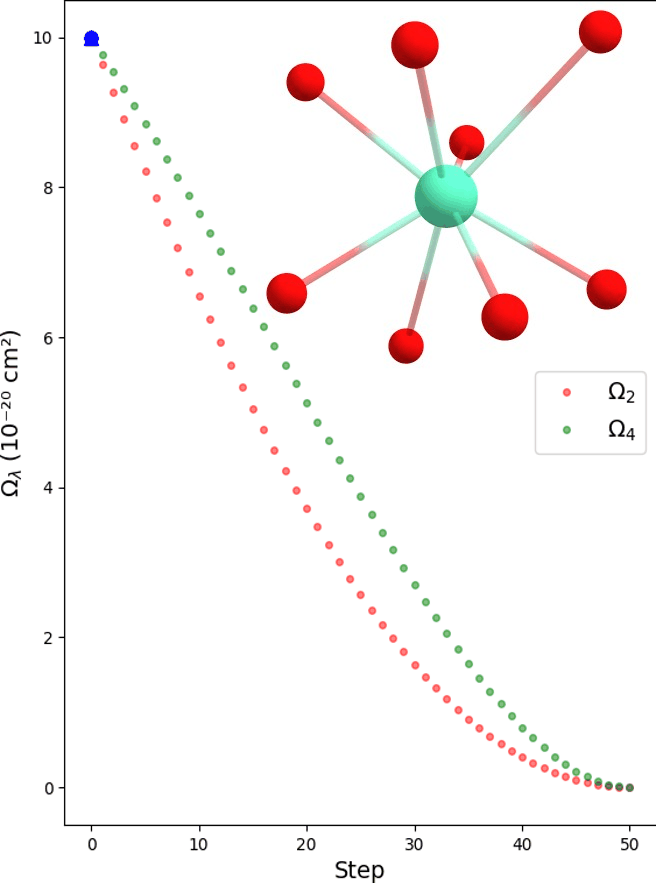

Supplement: Supplementary file 2 — Video [file SMLL-21-e08497-s001.gif]
